# Mass cytometric detection of homologous recombination proficiency in circulating tumor cells to predict chemoresistance of metastatic breast cancer patients

**DOI:** 10.1002/ijc.35498

**Published:** 2025-06-02

**Authors:** Kathrin Niedermayer, Henning Schäffler, Georgios Vlachos, Sara Greco, Kerstin Pfister, Barbara Volz, Leonie Ott, Hans Neubauer, Bernhard Polzer, André Koch, Sabine Riethdorf, Tanja Fehm, Wolfgang Janni, Thomas W. P. Friedl, Brigitte Rack, Ellen Heitzer, Fabienne Schochter, Lisa Wiesmüller

**Affiliations:** ^1^ Department of Obstetrics and Gynecology Ulm University Ulm Germany; ^2^ Institute of Human Genetics, Diagnostic & Research Center for Molecular BioMedicine Medical University of Graz Graz Austria; ^3^ Christian Doppler Laboratory for Liquid Biopsies for Early Detection of Cancer Medical University of Graz Graz Austria; ^4^ Research Institute for Women's Health University of Tübingen Tübingen Germany; ^5^ Institute of Tumor Biology University Medical Centre Hamburg‐Eppendorf Hamburg Germany; ^6^ Department of Obstetrics and Gynecology University Hospital Düsseldorf Düsseldorf Germany; ^7^ Center for Integrated Oncology (CIO Aachen, Bonn, Cologne) Duesseldorf Germany; ^8^ Division of Personalized Cancer Therapy Fraunhofer Institute of Toxicology and Experimental Medicine ITEM‐R Regensburg Germany

**Keywords:** CTC, HRD, mass cytometry, metastatic breast cancer

## Abstract

Circulating tumor cells (CTCs) can serve as a liquid biopsy to gain insight into treatment responses and metastatic recurrence. Due to their rarity, the analysis of CTCs is challenging and commonly based on immunomagnetic technologies using antibodies against EpCAM. This study used mass cytometry (CyTOF®) for the identification and characterization of CTCs from longitudinally monitored metastatic breast cancer (mBC) patients. Functional analysis focused on DNA damage responses, particularly the DNA repair pathway of homologous recombination (HR) validated in BC cells from the pleura. Fifty‐two blood samples from 13 mBC patients were collected for the enumeration of CTCs using CellSearch® technology, isolation of CTCs together with peripheral blood mononuclear cells (PBMCs) and of plasma. Cell‐free DNA (cfDNA) from plasma was analyzed by shallow genome sequencing to determine tumor fraction (TF) and HR deficiency (HRD). CTC/PBMC mixtures were phenotyped by CyTOF® using a panel of 13 antibodies including anti‐γH2AX, 53BP1, and RAD51. CyTOF® identified CTCs correlating with CellSearch®‐ and cfDNA‐based quantifications, detected DNA damage in CTCs, and the dynamics of their HR status during genotoxic therapies. Our study shows that CyTOF®‐based phenotyping of CTCs from mBC patients shows promise as a method to monitor tumor progression and HR proficiency in real time for the identification of chemoresistance.

## What's New?

Circulating tumor cells can offer insights into treatment responses and metastatic recurrence, but current analysis methods are limited. Here, the authors tested a newly developed mass cytometry panel comprising biomarkers such as γH2AX and RAD51 in longitudinally monitored metastatic breast cancer patients. They not only identified circulating tumor cells but also captured their dynamic homologous recombination status during genotoxic therapies, showing large concordance with homologous recombination deficiency estimation using circulating tumor DNA. The results demonstrate the promise of the multiparametric method for monitoring tumor progression and identifying chemoresistance.

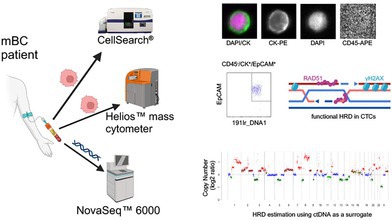

**Abbreviations**
BC: Breast Cancer
cfDNA: Cell‐free DNA
CSCs: cancer stem cells
CTCs: circulating tumor cells
ctDNA: circulating tumor DNA
CyTOF®: mass cytometry
DDRs: DNA damage responses
DSB: double‐strand break
EMT: epithelial‐mesenchymal transition
GTs: genotoxic therapies
HR: homologous recombination
HRD: HR deficiency
IF: immunofluorescence microscopy
LGAs: large genomic alterations
LOHs: loss‐of‐heterozygosities
LSTs: large‐scale State Transitions
mBC: metastatic breast cancer
MTi: microtubule inhibitory treatment
NHEJ: non‐homologous end joining
NT: no treatment
OS: overall survival
PARP: poly(ADP‐ribose)polymerase
PBMC: peripheral blood mononuclear cells
PE: Pleural effusion
PLD: Pegylated Liposomal Doxorubicin
sWGS: Shallow WGS
TF: tumor fraction
TNBC: triple‐negative breast cancer
WGS: whole exome/genome sequencing

## 1 INTRODUCTION

Breast Cancer (BC) is the most common malignancy and the leading cause of cancer deaths in women worldwide. CTCs can be detected in the bloodstream of 20%–30% of locally advanced BC patients as compared to >60% of patients with distant metastases.^1^ CTCs are shed from primary tumors, travel through the circulatory system, and disseminate to distant organs where they initiate the formation of metastases. In addition to CTCs, circulating tumor DNA (ctDNA) can be detected in the blood, consisting of small DNA fragments released into the bloodstream by tumor cells from various sources, including the primary tumor, metastases, and CTCs.^2^ Such CTCs and ctDNA have inspired the diagnostic concept of liquid biopsies, as they offer a way to track the state of metastatic seeds in real‐time.^3^ CTC counts have shown established value in prognosis and the prediction of treatment response, while CTC‐based predictions of therapeutic targets await final evidence for their clinical utility.^4^ Due to their rarity, the detection of CTCs is challenging and has been based on their biophysical or biological properties. The Food and Drug Administration has cleared the CellSearch® system for prognostic use in monitoring BC patients after initial therapy, which relies on positive selection of CTCs with EpCAM expression, which leads to a sub‐type bias and offers only one open fluorescence channel for biomarker detection.^5^

Genotoxic therapies (GTs) are still the predominating treatment type for mBC patients, especially of triple‐negative subtypes.^6^ Here, capturing multifactorial DNA damage responses (DDRs), DNA repair, and linked tumor cell phenotypes of relevance show promise, since altogether these features play a vital role in the plasticity of CTCs, their transition between epithelial and mesenchymal states, and the development of resistance mechanisms.^7^ Therefore, a technology that can identify and offer insight into the complex make‐up of CTC phenotypes is needed. CyTOF® can detect >40 antibodies simultaneously and is therefore potentially highly suited for providing information about disease signatures and therapy responses.^8^ Around one‐third of BCs exhibit defective HR, the most error‐free pathway of DNA double‐strand break (DSB) repair.^9^ DSBs arise during persistent replication stress, such as during GT with platinum‐based or poly(ADP‐ribose)polymerase (PARP)‐inhibitory drugs. Patients with HRD or germline *BRCA1/2* mutation status are likely sensitive to such treatments, which is why the determination of the HR status in mBC is important for treatment decisions.^10^, ^11^ Various HRD assays have been developed based either on genomic or functional characteristics. Genomic tests identify HRD‐specific mutational signatures or rearrangements like Large‐scale State Transitions (LSTs) by genome‐wide microarrays or whole exome/genome sequencing (WGS), that is, focusing on genomic scars that accumulate over time.^12^, ^13^ Functional assays have looked at the HR status in real‐time by measuring RAD51 foci formation in primary BC tissue or malignant pleural effusion (PE) after damage induction, which is measured by γH2AX foci formation through immunofluorescence microscopy (IF).^14^, ^15^

In this study, we (i) demonstrate unbiased detection of CTCs from longitudinally monitored mBC patients by use of CyTOF®, (ii) compare these results with CellSearch®‐ and shallow WGS‐based quantification of the TF, (iii) analyze CTC‐specific phenotypes, via RAD51 and γH2AX signals, in particular, and (iv) compare the resulting functional HRD status with ctDNA‐based, genomic HRD, and survival.

## 2 METHODS

### 2.1 Patient characteristics and sample collection

This study is part of a single‐center study on the longitudinal detection and analysis of CTCs in mBC patients. All patients treated for mBC between January 2017 and March 2024 at the Department of Gynecology and Obstetrics, University Hospital Ulm, were eligible for inclusion at the start of a new therapy, regardless of tumor biology and prior treatment. Further details of patient selection and sample collection as well as processing including PE samples are described in Önder et al.^16^ and in the Data S1 (methods section).

For ethics see Ethics Statement below.

### 2.2 Cell culture and treatment, CTC enrichment and enumeration by CellSearch® technology

Cell lines were cultivated as described in Schochter et al.^17^ (Data S1). The CTC enumeration and enrichment was performed as described in Schochter et al.^17^ The fourth channel was used for 53BP1 detection, and the 53BP1 score was calculated as described.

### 2.3 EDTA blood sample processing and mass cytometry

Two 7.5 mL EDTA blood samples were used for the isolation of cfDNA from plasma and PBMCs, together with CTCs. Frozen cells were thawed and then stained with the antibody panel shown in Tables S2 and S3. The samples were then analyzed using a Helios™ mass cytometer. Further details for isolation, staining, and data acquisition can be found in Data S1 (methods section).

### 2.4 Immunofluorescence microscopy

MCF‐7/182R‐6 and MDA‐MB‐436 cells were treated for 24 h with 10 μM Olaparib and stained with antibodies against γH2AX, RAD51, 53BP1, pRPA32, Cyclin A, EpCAM, Cytokeratin, and Vimentin as specified in Data S1 (methods section).

### 2.5 Cell‐free DNA isolation, shallow WGS library preparation, and sequencing

cfDNA was isolated from 2 mL plasma, retrieved from EDTA tubes of the same blood draw as the CellSave® tube used for CTC analysis and stored at −80°C. For cfDNA isolation, the QIAamp MinElute ccfDNA Mini Kit (50) (Qiagen, Venlo, Netherlands) was used according to the manufacturer's protocol. The cfDNA was then stored at −20°C.

Shallow WGS (sWGS) libraries were prepared with 10 ng of cfDNA using the plasma‐seq method as described.^18^ DNA quantity was determined using the Qubit™ dsDNA High Sensitivity Kit (Invitrogen, ThermoFisher Scientific, USA). Shotgun libraries were prepared with the TruSeq DNA Nano Sample Preparation Kit (Illumina, San Diego, CA). Final libraries were quantified and pooled equimolarly using the Qubit™ dsDNA High Sensitivity Kit and quality checked with the Agilent DNA 7500 kits (Agilent, Santa Clara). Pooled libraries were quantified via qPCR with the StepOne Real‐Time PCR System (Invitrogen, ThermoFisher Scientific, USA). Sixty libraries were pooled equimolarly and sequenced on an SP flow cell on a Novaseq 6000 system (Illumina, San Diego, CA) resulting in a ∼0.1–0.2× coverage per sample. The loading concentration was 1.1 nM and the samples were sequenced Paired End (PE 2x150bp).

The sequencing coverage and quality statistics for each sample are summarized in Table S7.

### 2.6 cfDNA data analysis, TF and HRD score estimation

The Illumina® Novaseq 6000 system provides the user with basecall files. Demultiplexing was performed on the Illumina® Dragen server after the run was finished. FASTQ files were trimmed using trimmomatic and quality checked with fastqc. Alignment was performed using the BWA‐MEM (version 0.7.9a‐r786) on the reference genome hg19/GRCh37.

The TF of each sample was determined using the ichorCNA pipeline.^19^

HRD scoring of PE samples was performed based on Telli et al.^20^ and as detailed in the Data S1 (methods section).

HRD scoring of the cfDNA from plasma was performed based on Eeckhoutte et al.^21^

## 3 RESULTS

### 3.1 Mass cytometric identification of CTCs from mBC patients

**FIGURE 1:**
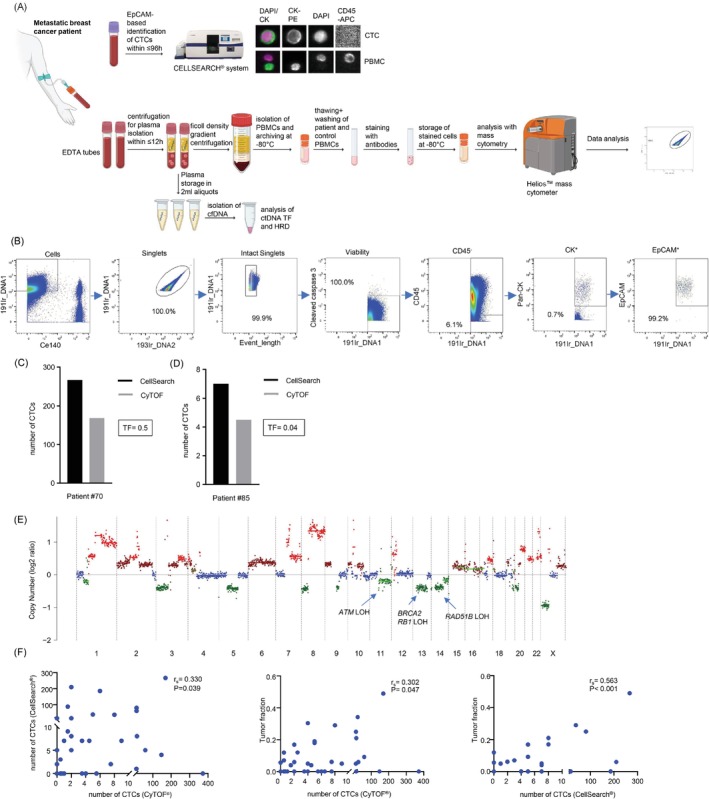
Plasma‐ and cell‐based analyses of liquid biopsies from mBC patients. (A) Workflow of CellSearch®, CyTOF®, and ctDNA analyses. (B) Gating strategy for CyTOF® data. The DNA marker ^191^Ir and ^140^Ce‐coupled beads are used to separate cells from beads. The DNA intercalating isotopes of Iridium ^191^Ir and ^193^Ir are used to select singlets. To remove remaining doublets, singlets are further gated on the Event Length parameter. Apoptotic cells were excluded by a cleaved caspase 3 gating. To detect CTCs, CD45^−^, CK^+^, and EpCAM^+^ gates are sequentially applied on live cells. (C, D) Enumeration of CTCs/7.5 mL blood by CellSearch® and CyTOF® and assessment of corresponding tumor fractions (TFs) for patients #70 and #85. CTCs are CD45^−^/panCK^+^/EpCAM^+^. (E) shallowHRD plot of patient #70 represents the normalized ratio of each segment compared to all the others. Each dot is a segment, and the segments at 0 are the ones that were not affected by copy number alterations. Highlighted are loss‐of‐heterozygosities (LOHs) of *ATM*, *BRCA2*, *RB1*, and *RAD51B*. (F) Correlations between the numbers of CTCs/7.5 mL blood identified by CellSearch® and CyTOF®, the number of CTCs/7.5 mL blood identified by CyTOF® and the respective TF, and the number of CTCs/7.5 mL blood identified with CellSearch and the respective TF. Given are the Spearman's rank correlation coefficients *r*_*s*_ and corresponding *P* values.

Aiming at establishing a multiparametric method for CTC identification and characterization in addition to the currently used CellSearch® system, we analyzed samples from a cohort of mBC patients before, during, and after systemic treatment (Table 1) engaging CyTOF®, whereby we compared the two methods (Figure 1A).^17^

**TABLE 1:** Summary of mBC patient characteristics together with CTC and cfDNA features from longitudinally collected blood samples.

Pat. No.	Primary tumor subtype	Metastasis subtype	Sample date (day.month.year)	Treatment status	Line of treatment in the metastatic setting	CTC number^a^ (CellSearch®)	CTC number^a^ (CyTOF®)	TF^b^	HRD^c^ (ctDNA®)	HRD^d^ (CyTOF®)	γH2AX^+^ (%)	RAD51^+^ (%)	RAD51^+^/γH2AX^+^ (%)	53BP1^+^ (%)	Cyclin A^+^ (%)	CD44^+^/CD24^−^ (%)	ALDH1A3^+^ (%)	Vimentin^+^ (%)	pRPA32^+^ (%)	53BP1 score^e^ (CellSearch®) (%)
# 07	TNBC	TNBC	26.01.2018	NT		17	2			YES	25.0	0.0	0.0	25.0	0.0	0.0	0.0	0.0		0.0
			22.02.2018	GT	5.	9	1.5			YES	33.3	0.0	0.0	33.3	0.0	0.0	0.0	0.0		0.0
			06.04.2018	GT		7	1			n.a.	0.0	0.0	0.0	50.0	0.0	0.0	0.0	0.0		0.0
# 11	HR+/HER2+	HR+/HER2+	17.10.2017	NT		89	1.5			n.a.	0.0	33.3	0.0	66.7	0.0	0.0	33.3	33.3	0.0	
			07.11.2017	GT	4.	2	0													
			19.12.2017	GT		16	0													56.3
			20.02.2018	GT		39	5			YES	10.0	0.0	0.0	50.0	0.0	0.0	10.0	0.0	0.0	20.5
# 20	TNBC	HER2+	05.02.2018	NT		0	0	0.056	NO										0.0	0.0
			26.02.2018	MTi	1.	0	0.5	0.000		n.a.	0.0	0.0	0.0	0.0	0.0	0.0	0.0	0.0	0.0	0.0
			19.03.2018	MTi		0	372.5	0.000		n.a.	9.2	4.3	46.4	36.6	2.2	1.7	0.4	10.6	30.7	0.0
			14.05.2018	MTi		0	1	0.000		n.a.	0.0	0.0	0.0	0.0	0.0	0.0	0.0	0.0	0.0	0.0
			04.06.2018	MTi		4	146.5	0.000		n.a.	5.2	0.0	0.0	60.4	4.4	0.0	0.3	1.0	1.7	0.0
			15.06.2018	MTi		186	6	0.000		YES	16.7	0.0	0.0	16.7	0.0	0.0	0.0	0.0	8.3	0.0
			25.07.2018	NT		5	0	0.000											0.0	0.0
			04.09.2018	NT		0	1	0.000		n.a.	0.0	0.0	0.0	0.0	0.0	0.0	0.0	0.0	0.0	0.0
			16.10.2018	MTi	2.	2	7.5	0.061	NO	n.a.	6.7	0.0	0.0	26.7	0.0	0.0	6.7	0.0	0.0	150.0
			07.03.2019	GT	4.	0	1	0.000		n.a.	0.0	0.0	0.0	0.0	0.0	0.0	0.0	0.0	0.0	0.0
# 46	HR+/HER2−	HR+/HER2−	14.03.2019	NT		5	59	0.092	NO	NO	16.9	4.2	5.0	71.2	7.6	0.0	0.0	8.5	15.3	0.0
			16.04.2019	NT		5	2	0.169	YES	YES	25.0	0.0	0.0	50.0	0.0	75.0	25.0	0.0	0.0	0.0
			25.02.2020	GT	4.		4	0.304	YES	YES	12.5	0.0	0.0	50.0	0.0	62.3	0.0	0.0	0.0	
			03.03.2020	GT			21.5	0.342	YES	YES	27.9	2.3	0.0	53.5	2.3	62.8	0.0	2.3	4.7	
# 49	HR+/HER2−	HR+/HER2−	16.04.2019	NT		0	0.67	0.000		n.a.	0.0	0.0	0.0	50.0	0.0	0.0	50.0	0.0	0.0	0.0
			07.05.2019	MTi	3.	0	3.5	0.000		n.a.	0.0	0.0	0.0	28.6	0.0	0.0	42.9	14.3	0.0	0.0
# 55	HR+/HER2−	TNBC	25.06.2019	NT		8	13.2	0.210	NO	n.a.	5.0	5.0	0.0	25.0	10.0	35.0	35.0	15.0	0.0	0.0
			12.11.2019	GT	2.		2.5	0.120	NO	YES	60.0	0.0	0.0	20.0	20.0	0.0	20.0	60.0	0.0	
			12.02.2020	GT	3.		5	0.180	NO	YES	60.0	0.0	0.0	10.0	0.0	20.0	0.0	0.0	0.0	
			16.03.2020	GT			5	0.190	NO	NO	40.0	20.0	50.0	10.0	20.0	20.0	0.0	30.0	0.0	
# 57	HR+/HER2−	TNBC	01.07.2019	NT		0	4	0.000		YES	83.3	0.0	0.0	0.0	16.7	0.0	0.0	66.7	0.0	0.0
			12.07.2019	NT		0	5.5	0.000		NO	27.3	9.1	33.3	0.0	0.0	0.0	18.2	54.6	0.0	0.0
			16.08.2019	GT	2.	0	3.5	0.000		NO	71.4	14.3	20.0	0.0	42.9	0.0	14.3	85.7	0.0	0.0
			21.02.2020	GT			6.5	0.000		YES	53.8	0.0	0.0	7.7	23.1	7.7	23.1	84.6	0.0	
# 61	TNBC	liver: HR+ skin: TNBC	05.11.2019	NT		210	2	0.060		YES	33.3	0.0	0.0	33.3	0.0	0.0	0.0	0.0	0.0	1.9
			26.11.2019	GT	3.		25	0.060		NO	52.0	16.0	11.5	58.0	12.0	0.0	8.0	30.0	50.0	
			17.12.2019	GT			1.5	0.040		YES	66.7	0.0	0.0	33.3	0.0	0.0	0.0	0.0	0.0	
			14.02.2020	GT	4.		2	0.000		YES	75.0	0.0	0.0	0.0	25.0	0.0	0.0	25.0	0.0	
			27.03.2020	GT			7.5	0.000		YES	20.0	0.0	0.0	0.0	6.7	0.0	20.0	53.3	0.0	
			21.08.2020	MTi	5.	16	2	0.000		YES	50.0	0.0	0.0	0.0	0.0	0.0	50.0	25.0	0.0	0.0
			11.09.2020	MTi			13	0.000		NO	46.2	3.9	8.3	0.0	3.8	7.7	15.4	57.7	0.0	
			02.10.2020	MTi		7	9	0.050		YES	27.8	0.0	0.0	5.6	5.6	0.0	16.7	55.6	0.0	57.1
# 62	TNBC	TNBC	08.04.2020	NT		38	8	0.290	Borderline	YES	25.0	0.0	0.0	16.7	0.0	0.0	8.3	58.3	25.0	0.0
			14.07.2020	GT	1.		1	0.000		n.a.	50.0	0.0	0.0	0.0	0.0	50.0	50.0	0.0	0.0	
			24.08.2020	NT		7	3.5	0.000		YES	14.3	14.3	0.0	14.3	42.9	0.0	0.0	71.4	0.0	57.1
# 70	TNBC	TNBC	22.12.2020	NT		267	168.7	0.490	Borderline	NO	34.0	16.9	14.0	1.2	13.4	0.0	0.4	16.6	1.6	7.9
#79	HR+/HER2−	HR+/HER2−	07.09.2021	NT		0	0.5	0.119	NO	n.a.	0.0	0.0	0.0	0.0	0.0	0.0	0.0	100.0	0.0	0.0
			07.10.2021	MTi	3.	0	0	0.000											0.0	0.0
			04.11.2021	MTi		1	10.5	0.049	NO	n.a.	9.5	0.0	0.0	47.6	0.0	0.0	9.5	2.3	19.0	0.0
# 85	TNBC	TNBC	17.12.2021	NT		7	4.5	0.040		YES	11.1	0.0	0.0	0.0	22.2	0.0	22.2	11.1	0.0	0.0
# 106	HR+/HER2−	TNBC	23.02.2023	NT		81	11.5	0.250	YES	n.a.	8.7	0.0	0.0	8.7	8.7	0.0	0.0	13.0	0.0	0.0
			23.03.2023	GT	1.		4	0.050		YES	25.0	0.0	0.0	12.5	0.0	0.0	37.5	25.0	0.0	0.0
			24.05.2023	NT		8	2	0.170	Borderline	YES	25.0	0.0	0.0	50.0	0.0	0.0	25.0	0.0	0.0	0.0
			14.06.2023	GT	2.	3	0.5	0.070		n.a.	0.0	0.0	0.0	0.0	0.0	0.0	0.0	0.0	0.0	0.0
			20.03.2024	GT	3.	62	12			YES	20.8	4.2	0.0	66.7	4.2	0.0	4.2	4.2		9.7

Abbreviations: GT, blood draw during genotoxic treatment (lilac shading) (Doxorubicin, Epirubicin, Carboplatin+nabPaclitaxel, Carboplatin+Gemcitabin, Carboplatin, Olaparib, Capecitabine+Lapatinib, Sacituzumab‐Govitecan); HER2+, human epidermal growth factor receptor positive; HR+, hormone receptor positive; HRD, Homologous Recombination Deficiency; MTi, blood draw during treatment with microtubule inhibitor (blue shading) (Vinorelbin+Trastuzumab+Pertuzumab, Eribulin, Paclitaxel+Bevacizumab, nabPaclitaxel, TDM‐1); n.a., not applicable regarding HRD (CyTOF®) categorization, if total number of CTCs<3 or RAD51^+^/γH2AX^+^ = 0 but γH2AX^+^ < 10%; NT, no treatment; Pat. No., patient number; TNBC, triple‐negative breast cancer.

^a^ CTC numbers are given per 7.5 ml blood, whereby a total volume of 7.5 ml blood was analyzed via CellSearch® and of at least 15 ml blood via CyTOF®.

^b^ TF, Tumor fraction based on ichorCNA providing an estimation of the amount of tumor‐derived genetic material present in the cfDNA sample. A fraction below 0.03 (3%) is not reliable.

^c^ HRD (ctDNA) metric is based on the detection of large genomic alterations (LGAs) in ctDNA, defined as breaks larger than 3 Mb that are within 10 Mb of each other. >20 LGAs identifies HRD (YES), 15–19 Borderline HRD (Borderline), 0–14 no HRD (NO).

^d^ HRD (CyTOF**®**) metric is based on the detection of γH2AX^+^ CTCs with RAD51^+^/γH2AX^+^ < 1.0% (YES) as compared to γH2AX+ CTCs with RAD51^+^/γH2AX^+^ ≥ 1.0% (NO). Concordance between HRD detection via ctDNA**®** (YES/Borderline) and CyTOF**®** (YES) is marked by green shading, discordance (NO) by red shading.

^e^ 53BP1 score (CellSearch®) encompassing the fraction of 53BP1‐positive CTCs and their 53BP1‐staining intensities is based on the formula described in Schochter et al.^21^

Moreover, EDTA tubes from the same blood draw were used to prepare plasma samples for cfDNA isolation. The cfDNA underwent sWGS to estimate the ctDNA fraction. The HRD phenotype was identified based on a metric similar to LSTs, focusing on large genomic alterations (LGAs), specifically breaks >3 Mb occurring within 10 Mb of each other. Between 0 and 14 LGAs signify no HRD, 15–19 borderline HRD, and >20 LGAs HRD.^21^

For CyTOF® identification of CTCs, a gating strategy was established (Figure 1B and Data S1 [methods section]). Apoptotic cells were excluded by a cleaved caspase 3 gating. To detect CTCs, the same antigens targeted in the CellSearch® system were used (CD45^−^, panCK^+^ and EpCAM^+^). To provide first proof‐of‐concept, a patient sample (#70) was analyzed, which according to CellSearch® contained 267 CTCs/7.5 mL whole blood. We enumerated 168.7 CTCs/7.5 mL via CyTOF® in 22.5 mL blood, therefore achieving a concordance between CellSearch® and CyTOF® of 63%. The TF was 0.5, and borderline HRD was detected in ctDNA (Figure 1C,E). Next, we analyzed a sample from a second patient (#85) with only 7 CTCs/7.5 mL blood identified by CellSearch®. Using CyTOF®, we counted 4.5 CTCs/7.5 mL whole blood in 15 mL blood, indicating a recovery rate of 64%. Matching lower CTC numbers, the TF reached only 0.04 (Figure 1D). These results suggested that CyTOF® enables reliable identification and quantification of CTCs, similar to CellSearch® results and in concordance with the determined TFs. This conclusion was supported by subsequent CyTOF® measurements in 13 patients, including pairwise comparisons between CyTOF and CellSearch® analysis in 31 blood samples and between CyTOF® and TF analysis in 44 blood samples, revealing statistically significant positive correlations among the data obtained with the three methods (Figure 1F).

For comparison, we also tested the identification of several other subsets of cells, that is, CD45^−^/EpCAM^+^, CD45^−^/panCK^+^, CD45^−^/panCK^−^/EpCAM^+^, CD45^+^/panCK^+^, CD45^−^/Vimentin^+^ and CD45^−^/Vimentin^+^/panCK^+^ (Table S1). Each one of these alternative marker combinations, except for CD45^−^/Vimentin^+^/panCK,^+^ detected cells in each patient blood sample, whereas the more stringent marker combination CD45^−^/panCK^+^/EpCAM^+^ failed to detect CTCs in some of the samples, as seen when using CellSearch® technology. Importantly, when performing Spearman correlation analyses, none of these alternative marker combinations resulted in cell numbers that would show a statistically significant correlation with CTC counts by CellSearch®. From these observations, we conclude that discrepancies between CellSearch®‐ and CyTOF® (CD45^−^/panCK^+^/EpCAM^+^)‐based counts of CTCs from the same blood draw (Table 1) are unlikely to be explained by different proportions of CK^+^, EpCAM^+^, or Vimentin^+^ cells and may rather point to technical aspects such as physical CTC enrichment (CellSearch®) versus detection in a blood‐derived cell mixture (CyTOF®), different affinities of antibodies used, or different detection principles (microscopy vs. mass cytometry). Importantly, CyTOF® analysis also enabled the exclusion of debris and dead cells by multiple parameters such as via cleaved caspase 3 gating. For further multiparametric phenotyping, we identified CTCs using the stringent CD45^−^/panCK^+^/EpCAM^+^ marker set, revealing cross‐correlations with CellSearch® and TF.

### 3.2 Detection of DNA damage responses in mBC cells by mass cytometry as compared to immunofluorescence microscopy

**FIGURE 2:**
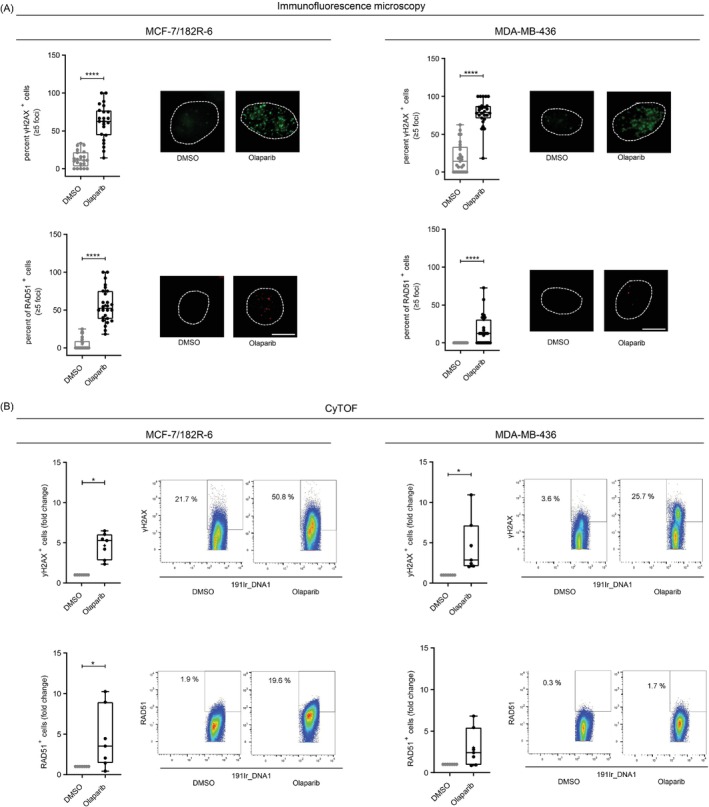
Comparing IF and CyTOF® as methods for the detection of DNA damage responses. (A) IF was performed with MCF‐7/182R‐6 and MDA‐MB‐436 after DMSO and Olaparib (10 μM) treatment for 24 h and γH2AX and RAD51 staining using fluorescently labeled antibodies. Cells were considered positive for RAD51 and γH2AX if they had ≥5 foci per cell. Between 10 and 15 images per experiment from two individual experiments were analyzed, respectively. Boxes, intraquartile range; horizontal lines, median; cross, mean; whiskers, min to max. Significances were calculated by Mann–Whitney *U* test using GraphPad Prism 9. ****P < 0.0001. Exemplary images are shown, scale bar: 10 μm. (B) CyTOF® was performed with MCF‐7/182R‐6 and MDA‐MB‐436 after DMSO and Olaparib (10 μM) treatment for 24 h and γH2AX and RAD51 staining with metal‐conjugated antibodies. For γH2AX^+^ and RAD51^+^ cells fold changes are given. Mean values from DMSO‐treated samples were set to 1 (absolute mean percentages for γH2AX^+^ MCF‐7/182R‐6 cells: 8.4%, MDA‐MB‐436 cells: 9.8%, for RAD51^+^ MCF‐7/182R‐6 cells: 5.2%, MDA‐MB‐436 cells: 1.9%). Boxes, intraquartile range; horizontal lines, median; cross, mean; whiskers, min to max. Significances were calculated by Wilcoxon test using GraphPad Prism 9. *P < 0.05. *N* = 6–7. Exemplary dot plots are shown.

To capture predictive and/or prognostic features of CTCs in the blood samples from mBC patients, we expanded our CyTOF® antibody panel by adding markers for DDRs and epithelial‐mesenchymal transition (EMT)^7^ (Tables S2 and S3). Proof of concept was provided using well‐characterized mBC cell lines, in this case MCF‐7/182R‐6 and MDA‐MB‐436 lines, to validate the method and the antibodies in a luminal and a triple‐negative breast cancer (TNBC) cell line, respectively. To this end, we directly compared the results from CyTOF® with IF, which so far is regarded as the gold standard for the analysis of DDRs. First, we comparatively analyzed epithelial (EpCAM, CK) and mesenchymal (Vimentin) cell markers (Figure S1). Underscoring the validity of CyTOF®‐based CTC detection, we obtained comparable results via IF and CyTOF® in MCF‐7/182R‐6 (EpCAM^+^, CK^+^, Vimentin^low^) and MDA‐MB‐436 cells (EpCAM^low/−^, CK^low^, Vimentin^+^). Second, we treated the cells with the PARP inhibitor Olaparib for 24 h to mimic GT of relevance for mBC patients (Table 1). PARP inhibitors cause replication stress, inducing nuclear accumulation of phosphorylated H2AX (γH2AX) and of RAD51 involved in the protection and reactivation of replication forks.^22^, ^23^ IF revealed significant increases of γH2AX^+^ and RAD51^+^ cells in both cell lines after Olaparib treatment (Figure 2A). γH2AX positivity reached a similar level in the two cell lines. MCF‐7/182R‐6 cells displayed an 11‐fold Olaparib‐induced increase in RAD51^+^ cells. MDA‐MB‐436 cells, carrying a deleterious *BRCA1* mutation and therefore HRD, showed negligibly low RAD51 signals before treatment and four‐fold (*p* < 0.0001) lower RAD51 values compared to MCF‐7/182R‐6 after treatment. Similar results were obtained with CyTOF® (Figure 2B). Again, we observed significantly more γH2AX^+^ cells after treatment of both lines. RAD51 positivity significantly increased in MCF‐7/182R‐6 cells (four‐fold), while the less pronounced increase in MDA‐MB‐436 cells did not reach statistical significance. Given that replication stress can prolong S‐phase, we further investigated the S‐/G2‐phase marker Cyclin A. Indeed, Olaparib treatment induced two‐ to four‐fold larger fractions of Cyclin A^+^ cells regardless of the method used and the cell line studied, though not reaching statistical significance in MDA‐MB‐436 using CyTOF® (Figure S1). However, IF‐based 53BP1 foci numbers rose two‐fold in MCF‐7/182R‐6 and in MDA‐MB‐436 post‐treatment, while according to CyTOF® only a 1.5‐fold augmentation was noticed in MDA‐MB‐436 and none in MCF‐7/182R‐6. Accumulation of the phosphorylated form of the human single‐stranded DNA‐binding protein RPA at Ser33 (pRPA32) serves to monitor the accumulation of ssDNA during replication stress.^24^ pRPA32^+^ cells were significantly elevated upon Olaparib treatment in MCF‐7/182R‐6 and MDA‐MB‐436 cells, while CyTOF® revealed such increase only in MCF‐7/182R‐6 cells. From this, CyTOF® analysis of γH2AX and RAD51 recapitulated IF of DDR markers most accurately, additionally showing promise to enable detection of HRD in CTCs by this method.

### 3.3 Monitoring genotoxic treatment responses in CTCs from mBC patients

**FIGURE 3:**
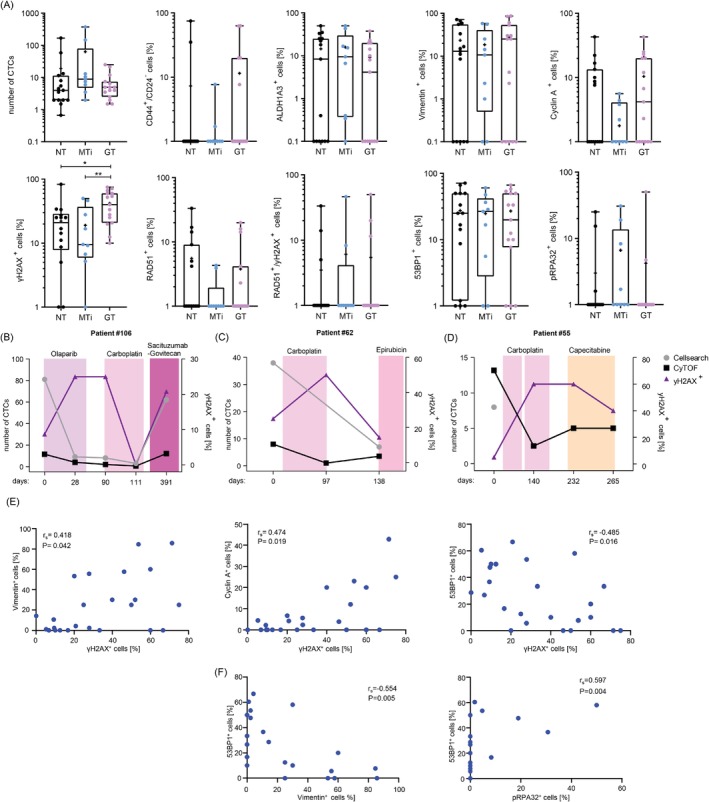
Analysis of CTC numbers and biomarker positivities under different treatment lines. (A) CyTOF®‐based enumeration of CD45^−^/panCK^+^/EpCAM^+^ CTCs/7.5 mL blood from mBC patients undergoing no treatment (NT), microtubule inhibitor treatment (MTi) or genotoxic treatment (GT), as well as further CTC characterization regarding the fractions of CD44^+^/CD24^−^, ALDH1A3^+^, Vimentin^+^, Cyclin A^+^, γH2AX^+^, RAD51^+^, RAD51^+^/γH2AX^+^, 53BP1^+^ and pRPA32^+^ CTCs in percent. Boxes, intraquartile range; horizontal lines, median; cross, mean; whiskers, min to max. Significances were calculated by Mann–Whitney *U* test, in case of statistical significance being reached with Kruskal–Wallis H‐test, using GraphPad Prism 9. *P < 0.05, **P < 0.01. (B–D) The numbers of CTCs/7.5 mL blood identified by CellSearch® (grey) or CyTOF® (black) are graphically displayed as well as the percentages of γH2AX^+^ CTCs (purple) identified by CyTOF®. Shown are data for samples from patients #106, #62 and #55. (E) Correlation analyses for γH2AX^+^ versus Vimentin^+^, Cyclin A^+^ and 53BP1^+^ CTCs. (F) Correlation analyses for 53BP1^+^ versus Vimentin^+^ and pRPA32^+^ CTCs. Spearman's rank correlation coefficients and *P* values were calculated in (E) and (F). For the analyses in (A), (E) and (F) only samples with ≥3 CTCs/15 mL blood were included.

Having demonstrated the validity of CyTOF® for identification of CTCs in blood‐derived cells and for detection of DDRs in mBC cell lines, we analyzed 52 blood samples longitudinally collected from 13 mBC patients by use of the same antibody panel designed for DDR analysis (Table S3). Recruited patients were treated with a variety of agents, which could be categorized as either microtubule inhibitory or genotoxic according to their mode‐of‐actions (Table 1). Having found that mBC cells showed significantly elevated γH2AX‐positivities in response to PARP inhibition (Figure 2B), we evaluated GT‐dependent changes by applying our DDR CyTOF® panel on samples where at least 3 CTC/15 mL blood were detected as CD45^−^ /panCK^+^/EpCAM^+^ (Figure 3A). We did not observe differences in the median CTC numbers during GT compared to no treatment (NT) or MTi. This was also true when focusing on CTCs showing positivity for stemness markers (CD44^+^/CD24^−^, ALDH1A3^+^) or signs of EMT (Vimentin^+^). The median percentage of Cyclin A^+^ CTCs showed a non‐significant trend to be lower during MTi compared to GT (*p* = 0.1478), in line with the G2‐ or M‐phase arrest known to be caused by MTi treatment. Importantly, we saw a significant two‐fold increase in the percentage of γH2AX^+^ CTCs during GT compared to NT or MTi, while RAD51^+^, 53BP1^+^, and pRPA32^+^ CTCs did not differ significantly among these treatment groups. The same analysis was performed with CD45^−^/EpCAM^+^ and CD45^−^/panCK^+^ cells (Figure S2 and Tables S4 and S5). CD45^−^/EpCAM^+^ cells showed a significant increase in the percentage of γH2AX^+^ cells during GT compared to NT or MTi, and CD45^−^/panCK^+^ cells showed a significant increase in the percentage of γH2AX^+^ cells during GT and NT compared to MTi. In CD45^−^/panCK^+^ cells, the decrease in Cyclin A^+^ cells during MTi treatment compared to NT and GT reached statistical significance.

Examples of patients showing increases in γH2AX^+^ CTCs under different lines of GT are shown in Figure 3B–D. The graphs depicting longitudinal analysis of the markers γH2AX^+^, RAD51^+^/γH2AX^+^ and 53BP1^+^ for these three and six additional mBC patients are shown in Figures S3 and S4. Notably, graphs for patients #49 and #79 exclusively undergoing MTi visualize that in CTCs from these patients, γH2AX‐positivity was detectable in one out of four samples and in <10% of CTCs only, but 53BP1‐positivity in three out of four samples with up to 50% of CTCs. 53BP1 (CyTOF®) signals were considered, as identified in 68% of all tested samples as compared to only 21% using CellSearch® (Table 1). From these observations, γH2AX signals in CTCs feature phenotypic changes caused by GT, that is, accumulation of DNA damage and its removal by DNA repair. For comparison, 53BP1 signals in CTCs may rather be informative for mBC patients during MTi, where 53BP1 associates with loss of genomic integrity, as previously demonstrated by single‐cell PCR analysis.^17^

To unveil possible connections between DNA damage, indicated by γH2AX‐positivity, stemness, EMT, and/or DNA repair in CTCs, we performed Spearman correlation analyses for γH2AX versus all other CyTOF® biomarker values (Figure 3E and Table 2). Such analyses revealed significant positive correlations with Vimentin and Cyclin A values. These findings are compatible with a prolongation of S‐phase due to damage‐induced replication stress, which induces EMT.^7^ Furthermore, we noticed a significant negative correlation between γH2AX‐ and 53BP1‐positivity. This led us to correlate 53BP1‐positivity with all other markers, indicating significant negative correlation with Vimentin and positive correlation with pRPA32‐positivity (Figure 3F and Table 2). Our data suggest the existence of two CTC populations with opposed phenotypes characterized by epithelial features and 53BP1‐signals as opposed to CTCs undergoing EMT and displaying γH2AX‐labeled DNA damage in S‐phase.

**TABLE 2:** Spearman correlation analyses of CTC (CyTOF®) marker stainings and survival.

	ALDH1A3^+^	53BP1^+^	γH2AX^+^	RAD51^+^	CD44^+^/CD24^−^	Vimentin^+^	Cyclin A^+^	pRPA32^+^
ALDH1A3^+^		*r*_*s*_ = −0.357 *p* = 0.087	*r*_*s*_ = −0.082 *p* = 0.702	*r*_*s*_ = −0.156 *p* = 0.468	*r*_*s*_ = −0.351 *p* = 0.093	*r*_*s*_ = 0.614 *p* = 0.001	*r*_*s*_ = 0.108 *p* = 0.615	*r*_*s*_ = −0.310 *p* = 0.160
53BP1^+^			*r*_*s*_ = −0.485 *p* = 0.016	*r*_*s*_ = 0.121 *p* = 0.572	*r*_*s*_ = 0.009 *p* = 0.966	*r*_*s*_ = −0.554 *p* = 0.005	*r*_*s*_ = −0.302 *p* = 0.151	*r*_*s*_ = 0.597 *p* = 0.003
γH2AX^+^				*r*_*s*_ = 0.174 *p* = 0.415	*r*_*s*_ = 0.098 *p* = 0.649	*r*_*s*_ = 0.418 *p* = 0.042	*r*_*s*_ = 0.474 *p* = 0.019	*r*_*s*_ = −0.317 *p* = 0.150
RAD51^+^					*r*_*s*_ = 0.326 *p* = 0.120	*r*_*s*_ = 0.362 *p* = 0.082	*r*_*s*_ = 0.457 *p* = 0.025	*r*_*s*_ = 0.335 *p* = 0.128
Survival (days)	*r*_*s*_ = 0.731 *p* = 0.049	*r*_*s*_ = −0.595 *p* = 0.132	*r*_*s*_ = 0.095 *p* = 0.840	*r*_*s*_ = −0.682 *p* = 0.077	*r*_*s*_ = 0.082 *p* = 0.875	*r*_*s*_ = 0.491 *p* = 0.221	*r*_*s*_ = 0.122 *p* = 0.794	*r*_*s*_ = −0.178 *p* = 0.714

*Note*: *r*_*s*_, Spearman's rho correlation coefficient; *P* < 0.05 is marked by dark green shading, *p* < 0.1 by light green shading; Survival: *n* = 8 (pRPA32 *n* = 7); markers: *n* = 24 (pRPA32 *n* = 22).

### 3.4 HR status, determined via genomic alterations in ctDNA and RAD51‐positivity in γH2AX ^+^ CTCs from mBC patients, changes dynamically upon administration of GT

**FIGURE 4:**
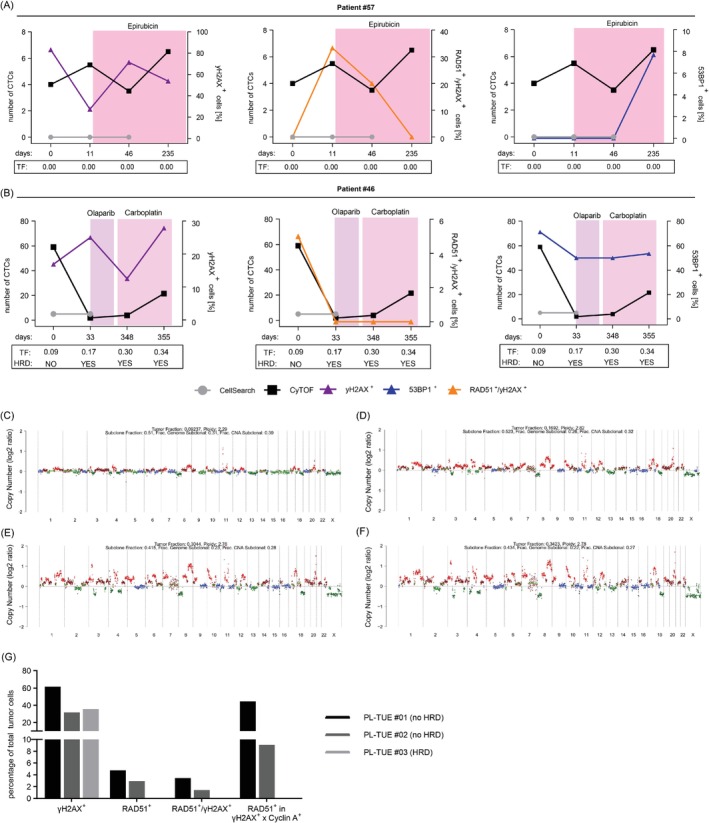
CyTOF®‐ and ctDNA‐based analysis of HRD. The panels graphically display the numbers of CTCs/7.5 mL blood identified by CellSearch® (grey) or CyTOF® (black), γH2AX^+^ (purple), RAD51^+^/γH2AX^+^ (orange) and 53BP1^+^ (blue) CTCs identified by CyTOF® in percent. CTCs are CD45^−^/CK^+^/EpCAM^+^. Below the panels TFs are given and in case of positivity (patient #46) also HRD status. Shown are data for samples from patient #57 (A) and #46 (B). ShallowHRD plots for each of the four samples from patient #46 are shown in (C), (D), (E) and (F). (G) Analysis of three pleura samples, that is, PL‐TUE #01 (no HRD) in black, PL‐TUE #02 (no HRD) in dark grey and PL‐TUE #03 (HRD) in light grey. Shown are the percentages of total tumor cells which are γH2AX^+^, RAD51^+^, RAD51^+^/γH2AX^+^ and RAD51^+^/γH2AX^+^ × Cyclin A^+^. For further information see Table S6.

Since we were particularly interested in monitoring the HR status, we focused on γH2AX and RAD51 CyTOF® analyses to gain insight into this pathway in CTCs. As an antagonist of HR and a promoter of non‐homologous end joining (NHEJ) we also analyzed 53BP1 in the same CTCs.^25^ As representatively shown in Figure 4 for two patients undergoing GT with longitudinal sampling for 12 months, we determined the percentage of γH2AX^+^ and 53BP1^+^ CTCs, as well as RAD51^+^/γH2AX^+^ CTCs, that is, the percentage of RAD51^+^ among γH2AX^+^ CTCs (Figures S3 and S4 and Table 1). These RAD51^+^/γH2AX^+^ CTCs, rather than mere RAD51 signals, served as a functional marker of HR proficiency to correct RAD51 functionality for DNA damage status in each single CTC individually.

Patient #57 (undergoing Epirubicin treatment) showed similar numbers of CTCs with γH2AX‐positivity in at least 27% of the cells throughout the observation period (Figure 4A). In contrast, administration of Epirubicin was accompanied by a steady decline of the RAD51/γH2AX double‐positive fraction of CTCs from 33% before the onset of treatment down to 0%. Of note, 53BP1‐positivity could only be detected at the end of Epirubicin treatment, precisely when RAD51^+^/γH2AX^+^ CTCs disappeared. In all patient #57 samples, showing 7–13 CTCs/15 mL blood according to CyTOF®, TFs were below the detection limit. For patient #46, we saw DNA damage in all samples indicated by γH2AX‐positivity in 13%–28% of the CTCs throughout longitudinal sampling (Figure 4B). However, while RAD51^+^/γH2AX^+^ CTCs were detected only before the sequential treatments with Olaparib and Carboplatin, 53BP1‐positivity remained high at 50%–71% during the whole observation period. The TF rose steadily during longitudinal sampling, correlating with an increase in CTC numbers starting from Olaparib administration (4–43 CTCs/15 mL blood), even though it was delayed when compared to the high CTC value before treatment (118 CTCs/15 mL blood). Most interestingly, accumulation of genomic alterations indicative of HRD (Figure 4C–F) did match the disappearance of RAD51^+^/γH2AX^+^ CTCs after the first sample.

To further validate the HR score determined by CyTOF®, we analyzed the tumor cells from three PE samples of BC patients after classical HRD scoring using genomic DNA from tumor samples (Figure 4G and Table S6). All three PE samples revealed γH2AX‐positivity in tumor cells, but only in samples from patients PL‐TUE #01 (no HRD) and PL‐TUE #02 (no HRD) we also detect RAD51‐positivity in tumor cells showing signs of DNA damage (RAD51^+^/γH2AX^+^) while residing in S/G2‐phase (RAD51^+^/γH2AX^+^ x CyclinA^+^). To the contrary, PL‐TUE #03 with known HRD did not show RAD51‐positivity.

Altogether, these studies suggest that CyTOF®‐based analyses of DDRs, HR in particular, can detect a dynamic shift in DNA repair pathway usage in CTCs from mBC patients undergoing GT.

### 3.5 Correlations of CTC‐based ALDH1A3 and RAD51 signals with survival of mBC patients during treatment

Since both genomic as well as functional scores predictive of HRD in tumor tissues have been explored to predict responses of BC patients to platinum‐based, PARP‐inhibitory, and other genotoxic drugs,^15^, ^26^, ^27^ we determined if there were any correlations between our CyTOF®‐based markers and overall survival (OS) of the patients (Table 2). Thus, survival times of mBC patients between the date of the last blood sampling during systemic treatment and the date of death were calculated and plotted against the percentages of marker‐positive CTCs. Only CyTOF®‐analyzed samples with at least 3 CTCs/15 mL blood, drawn during the last treatment line, were included. We found a significant positive correlation between ALDH1A3^+^ CTCs and survival times, indicating that patients with CTCs expressing this stemness marker during the last analyzed treatment survived longer (Figure S5A). We also noticed a trend towards a negative correlation between RAD51^+^ CTCs and survival times, suggesting longer survival of patients with CTCs showing functional HRD (Table 2). Other CyTOF®‐based markers failed to correlate with survival times, which was also true for total numbers of CTCs identified by CellSearch® (*p* = 0.500) or CyTOF® (*p* = 0.327) without phenotypic subtyping (Table 2) as well as for the TFs (*p* = 0.119).

These ALDH1A3^+^ and RAD51^+^ CTC‐specific survival data led us to further investigate possible links between these markers and other features of CTCs (Figure S5B and Table 2). Percentages of ALDH1A3^+^ CTCs showed a significant positive correlation with Vimentin‐specific values, revealing an association between stemness and EMT in the analyzed CTCs. Percentages of RAD51^+^ CTCs showed a significant positive correlation with Cyclin A and a trend with Vimentin values, suggesting that RAD51 is expressed in CTCs during S‐Phase, particularly after the acquisition of mesenchymal features. All‐in‐all, we could see correlations between CyTOF®‐based CTC characteristics with each other as well as with survival times, revealing its potential as a tool to study the heterogeneity and evolution of CTCs and as a biomarker of therapy responses.

## 4 DISCUSSION

This study demonstrates the feasibility of CyTOF® as a multiparametric method for the identification and characterization of CTCs from mBC patients. Importantly, this methodology enables the analysis of single cells that we engage here to capture the HRD status of CTCs in real‐time, aiming at better prediction of treatment responses.

The current gold‐standard for CTC identification is the CellSearch® technology, which uses ferrofluidic EpCAM selection, but has only one free fluorescence channel for biomarker analysis. CyTOF® identified CTCs (threshold: ≥3 CTCs/15 mL) in 81% (21/26) of blood draws with CTC‐positivity by CellSearch® (threshold: ≥1 CTC/7.5 mL) and in 88% (23/26) of TF^+^ samples, indicating high sensitivity of our new CyTOF®‐based approach (Table 1). Of note, four blood draws with CTC‐positivity by CyTOF® were devoid of TF but in line with CellSearch® evaluations, supporting highly sensitive detection of CTCs independently of the method used. We also noticed CTC‐positivity by CyTOF® in five blood draws with no CellSearch®‐based CTC detection and differences in absolute CTC numbers that could be due to several reasons, for example, different affinities of antibodies used for identification of CD45^−^/panCK^+^/EpCAM^+^ CTCs, sample preparation or staining procedures (Figure 1A). Providing evidence for detection of tumor cells by CyTOF® and CellSearch® with comparable specificities, we calculated similar correlation coefficients when comparing either CTC count with the TF value, reflecting the amount of tumor‐derived genetic material in the patients' plasma from the same blood draw. Overall, detection by all three methods correlated significantly, underscoring their validities.

Proteomic identification of biomarkers in single CTCs has remained a challenge and was the main goal of this project. Here, we aimed at the development of an assay for functional HRD in CTCs based on DDR signals, of the core HR enzyme RAD51, in particular.^28^ Using mBC cell lines with defined HRD status, we established that DDR‐based biomarkers showed comparable results in CyTOF® and IF, the common method of their analysis. This was especially important for γH2AX and RAD51, as they serve as markers used in functional HRD tests.^14^, ^15^ Accordingly, RAD51 accumulation after Olaparib treatment was less pronounced in MDA‐MB‐436 cells with defined HRD, despite comparable γH2AX‐DNA damage levels (Figure 2B).

Given that the fragility of patient‐derived CTCs precludes routine ex vivo cultivation and ex vivo functional testing,^29^ we exploited in vivo DDRs in CTCs of patients undergoing GT. Therapeutically active compounds included platinum derivatives and PARP inhibitors, for which genomic and/or functional HRD have been shown to predict responses in BC patients,^6^, ^10^, ^11^, ^15^ anthracyclines, for which sensitivities were reported in BRCA1/BRCA2‐deficient tumors^30^ as well as DNA synthesis inhibitors, since recent research has carved out critical roles of HR components in DNA replication fork protection and reactivation.^23^ As expected, we saw a GT‐dependent increase in DNA damage, with significantly more γH2AX^+^ CTCs from mBC patients undergoing GT. Fractions of γH2AX^+^ CTCs ranged between 10% and 80% post‐GT, similarly to previously seen by CellSearch® in CTCs from two advanced refractory BC patients treated with Cyclophosphamide and PARP inhibitor.^31^ In fact, γH2AX was the only biomarker in our panel showing a significant change between the medians of the values for the different treatment groups.

Since both γH2AX and 53BP1 have classically been engaged as DNA damage markers, it was surprising to find that the percentages of γH2AX^+^ and 53BP1^+^ CTCs showed a negative correlation (Table 2). However, this observation can be explained by previously published quantitative image‐based cytometry data^32^ revealing that 53BP1 accumulates around DSBs primarily in G1‐phase. Our cross‐correlations revealed an association between γH2AX^+^ CTCs and Cyclin A‐positivity, suggesting a cell cycle arrest in S‐/G2‐phase post‐GT as previously seen in cancer cells^33^ and in line with data demonstrating that γH2AX not only detects DSBs but also other types of DNA damage such as replication stress.^22^ 53BP1 protects DNA ends from resection and untimely homology‐directed repair, particularly in HRD cells,^25^ and such antagonistic relationship might also be reflected by the opposing trends of RAD51^+^/γH2AX^+^ (or RAD51^+^) and 53BP1^+^ CTCs seen in patient #57 during Epirubicin treatment.

CTCs undergo EMT to enter the bloodstream, and the majority of CTCs were positive for the EMT marker Vimentin, reassuring that CyTOF®‐based CTC detection captures mBC cells presenting mesenchymal features. Interestingly, percentages of γH2AX^+^ and Vimentin^+^ CTCs showed a positive correlation, which strengthens the concept that DNA damage signaling, for example, via the upstream kinases ATM and ATR phosphorylating H2AX, stimulate EMT.^7^ Vimentin and RAD51 expression also showed a trend to positively correlate, in line with the fact that RAD51 is coupled with EMT in breast and prostate cancer.^7^ Vice versa, EMT has been reported to promote DDRs, triggering a vicious cycle of invasiveness and chemoresistance mechanisms.^7^ Further evidence for different phenotypes of γH2AX^+^ and 53BP1^+^ CTCs was provided by a negative correlation between 53BP1‐ and Vimentin‐positivities. Consistently, 53BP1 was reported to suppress EMT and therefore Vimentin expression.^34^ Such a negative correlation was also noticeable between 53BP1^+^ CTCs and CTCs expressing the stemness marker ALDH1A3 (Table 2), consistent with recent data showing fewer 53BP1 foci in ALDH1^+^ compared to ALDH1^−^ cells after irradiation.^35^ ALDH1A3, the isoform of ALDH1 which reflects most of ALDH activity in BC,^36^ has been associated with cell migration and metastasis.^37^ Previous study showed that ALDH1A3 induces mesenchymal differentiation in glioblastoma.^38^ We found that ALDH1A3 positively correlates with Vimentin, which altogether suggests that 53BP1^−^ CTCs are endowed with mesenchymal and stem cell features enabling them to migrate through the circulatory system to then disseminate and form metastases. Dynamic changes of not only DDR but also stemness markers, as seen here with patient‐derived CTCs, therefore support the idea that cancer stem cells (CSCs) are not a fixed entity, but rather that CSCness is a state that cells can enter.

One of the hallmarks of cancer is genomic instability. Strikingly, a fraction of up to 40%–70% of TNBCs carry mutations in a panel of HR repair pathway genes or feature *BRCA1* promoter methylation, resulting in a condition known as genomic scars or HRD.^11^, ^39^ Such mutational signatures were established to enlarge the fraction of primary BC as well as mBC with genomic HRD from 1% to 5% based on inherited *BRCA1*/*BRCA2* mutations to 20%–30%.^9^, ^13^ The power of genomic HRD to predict GT responses of TNBC, which has been associated with BRCAness, EMT, stem cell features, and metastasis,^40^ has been studied intensively. Ter Brugge and colleagues^10^ found HRD based on shallow WGS to predict the platinum response in ≤72% of patient‐derived TNBC xenografts. Genomic HRD was also reported to be associated with carboplatin response and survival in a study involving 225 early TNBC patients.^41^ However, BRCA‐proficient mBC with a high HRD score did not seem to benefit from platinum‐based chemotherapy regardless of molecular subtype,^6^ and only a small subset of mBC patients with high LOH scores without germline *BRCA1*/*2* mutation could benefit from PARP inhibition with Rucaparib.^42^ These findings underscored the need for additional biomarkers to guide platinum/PARP inhibitor use in mBC. Our study included three PE samples from BC patients with pre‐determined genomic HRD status, which was concordant with the respective HRD(CyTOF) status each (Figure 4G and Table S6), suggesting that HRD(CyTOF) may serve as such an additional biomarker, which complements genomic signatures by the treatment‐relevant phenotype of mBC.

In this context, it is important to note that DNA‐based tests provide a snapshot from the past by looking at mutagenic events that preceded the evolution of the tumor, which is subject to selection pressures during metastasis and treatment. Functional tests have the advantage to monitor the real‐time HRD status.^15^ In this study we compared genomic HRD determined via shallow sequencing of ctDNA and functional HRD determined via RAD51 and γH2AX CyTOF® scores in CTCs from the same blood samples. All‐in‐all, we monitored HRD(CyTOF) in 77% of samples (23/30), which is reminiscent of a recent study with 79% of TNBC biopsies showing RAD51^low^ scores.^11^ For comparison, only 44% of our TF^+^ samples (7/16) showed HRD(ctDNA), which lies in the range of genomic HRD scores of 44% in BC^13^ or 59% in TNBC.^11^ HRD(ctDNA) and HRD(CyTOF) categorizations were concordant in seven out of 10 samples, that is, in 70% of the cases passing both quality thresholds (Table 1), which is similar to the published concordance of 78% in xenografts.^10^ Among the three discordant samples one was derived from TNBC patient #70 without HRD(CyTOF) but borderline HRD(ctDNA). In this case, possible explanations for discordant categorization might be related to the observed LOHs in *BRCA2* and *ATM* (Figure 1E), because reduced levels of BRCA1/2 or ATM were reported to still support HR functions but derepress error‐prone homology‐directed DSB repair pathways generating genomic scars.^10^, ^43^, ^44^, ^45^ The two other samples with discordances were obtained from patient #55. In CTCs from this patient we monitored disappearance of HRD(CyTOF) during GT, while HRD(ctDNA) was absent throughout. This observation is in accordance with previous studies revealing that the functional HRD status in BC undergoes changes during GT.^10^ Dynamic HRD(CyTOF) changes were also observed in longitudinal samples from patients #57 and #61 as well as from patient #46, in the latter equally affecting HRD(ctDNA). In line with HRD(CyTOF) changes, patients #55, #57, and #61 also featured different BC subtypes at least at one metastatic site compared to the primary tumor, reflecting tumor evolution from the primary tumor to the mBC. Our observations strengthen the idea that HRD(CyTOF) as a functional marker may capture the current HRD phenotype in real‐time and therefore dynamic changes of the same. Genomic instability in tumor cells, that in this study is reflected by ctDNA, indicates that the tumor either is HRD or has been HRD in the past, possibly before resistant subclones emerged. ctDNA may also capture the HR status from tumor cell populations without EpCAM expression, which could explain the concordant absence of HRD(ctDNA) and of HRD(CyTOF) in cells from patient #55 when gating via CD45^−^/panCK^+^ rather than via the stringent CTC marker combination CD45^−^/panCK^+^/EpCAM^+^ (Tables 1 and S5). Ultimately, these findings suggest that a combination of genomic and functional HRD tests should be employed to ascertain the HRD status in CTCs.

Lastly, we found that the presence of ALDH1A3^+^ CTCs positively correlates with the survival of the treated mBC patients. ALDH1A3 was reported to be expressed in CTCs having acquired CSC and mesenchymal features^46^ and is commonly associated with chemoresistance and worse survival in many cancers including mBC, though only at baseline and no longer when monitored during chemotherapy.^47^, ^48^, ^49^ Notably, ALDH1A3 plays a non‐enzymatic role in drug detoxification, namely through gene expression changes and activation of signaling pathways such as the PI3K/AKT/mTOR pathway. However, ALDH1A3 has also been connected with better outcomes in *TP53* wildtype ovarian cancer, so that its effects might also be cell type‐, context‐, and treatment‐dependent.^49^ Conspicuously, our data further suggest an association of RAD51 expression in CTCs towards the end of treatment with worse survival, which is in accordance with literature data revealing high *RAD51* mRNA expression in BC, particularly in TNBC and advanced stages with poor prognosis.^50^ These findings highlight the potential of RAD51 overexpression as a chemoresistance mechanism.^28^ However, for a better understanding of the prognostic and predictive values of ALDH1A3‐ and RAD51‐positivity in CTCs, larger patient cohorts will have to be involved in future studies. In conclusion, we could show that CyTOF® is a valuable method not only to count CTCs but also to monitor dynamic changes in their DNA repair and differentiation status in response to administration of genotoxic drugs.

## AUTHOR CONTRIBUTIONS

**Kathrin Niedermayer:** Investigation; validation; formal analysis; visualization; writing – original draft; writing – review and editing; methodology. **Henning Schäffler:** Data curation; formal analysis; writing – review and editing; validation. **Georgios Vlachos:** Methodology; data curation; investigation; formal analysis; writing – review and editing; visualization. **Sara Greco:** Investigation; writing – review and editing. **Kerstin Pfister:** Data curation; writing – review and editing. **Barbara Volz:** Methodology; data curation; writing – review and editing. **Leonie Ott:** Methodology; writing – review and editing. **Hans Neubauer:** Data curation; formal analysis; writing – review and editing; project administration; funding acquisition. **Bernhard Polzer:** Data curation; formal analysis; writing – review and editing; project administration; funding acquisition. **André Koch:** Data curation; methodology; supervision; formal analysis; writing – review and editing; project administration; funding acquisition. **Sabine Riethdorf:** Supervision; methodology; formal analysis; writing – review and editing; project administration; funding acquisition. **Tanja Fehm:** Data curation; writing – review and editing; project administration; funding acquisition. **Wolfgang Janni:** Data curation; writing – review and editing; project administration; funding acquisition. **Thomas W. P. Friedl:** Data curation; formal analysis; writing – review and editing; project administration; funding acquisition. **Brigitte Rack:** Data curation; writing – review and editing; project administration; funding acquisition. **Ellen Heitzer:** Methodology; formal analysis; data curation; supervision; writing – review and editing. **Fabienne Schochter:** Conceptualization; supervision; data curation; writing – review and editing. **Lisa Wiesmüller:** Conceptualization; methodology; investigation; validation; formal analysis; supervision; funding acquisition; visualization; project administration; resources; writing – review and editing.

## FUNDING INFORMATION

This project was financially supported by the Deutsche Forschungsgemeinschaft (DFG, Research Training Group 2544, project B03, to Lisa Wiesmüller) and by the German Cancer Aid, Priority Program ‘Translational Oncology’ 70112504 (projects S1 to Wolfgang Janni, Thomas W. P. Friedl and Lisa Wiesmüller, S2 to Brigitte Rack, S3 to Tanja Fehm and Hans Neubauer, S5 to Sabine Riethdorf and S6 to Bernhard Polzer) and 70114705 (project S2 to Wolfgang Janni, Thomas W. P. Friedl, Brigitte Rack and Lisa Wiesmüller, S3 to Tanja Fehm and Hans Neubauer, S4 to André Koch, S5 to Sabine Riethdorf and S6 to Bernhard Polzer). Kerstin Pfister received Start‐up Funding in the Basic Clinician Scientist Program (Basic CSP) of the Medical Faculty of Ulm University. Kathrin Niedermayer and Sara Greco are members of the International Graduate School of Molecular Medicine of the Medical Faculty of Ulm University.

## CONFLICT OF INTEREST STATEMENT

All authors declare that they have no conflict of interest.

## ETHICS STATEMENT

The study was conducted in compliance with the guidelines of the Declaration of Helsinki and approved by the Ethics Committees of the Eberhard Karl University of Tübingen (protocol codes 150/2018BO2 and 288/2022BO2) and from the Ethics Committee of Ulm University Hospital (protocol code 20/17). Informed written consent was obtained from all participants. Study participation did not influence the patients' treatment. Participants were not informed about any results of CTC analysis.

## Supporting information


**DATA S1:** Supporting Information.

**TABLE S7:** Percentage of targeted bases with coverage ≥1.

## Data Availability

All data of this study will be made available upon request.
