# Supplementary material for: Mass cytometric detection of homologous recombination proficiency in circulating tumor cells to predict chemoresistance of metastatic breast cancer patients
Source: Int J Cancer. 2025 Jun 2;157(7):1465–80. doi: 10.1002/ijc.35498 (PMC12334908; doi:10.1002/ijc.35498)
Supplement: Supplementary file 1 — DATA S1: Supporting Information. [file IJC-157-1465-s001.pdf]

SUPPLEMENTARY MATERIAL

Mass cytometric detection of homologous recombination proficiency in circulating tumor cells to predict chemoresistance of metastatic breast cancer patients

Kathrin Niedermayer, Henning Schäffler, Georgios Vlachos, Sara Greco, Kerstin Pfister, Barbara Volz, Leonie Ott, Hans Neubauer, Bernhard Polzer, André Koch, Sabine Riethdorf, Tanja Fehm, Wolfgang Janni, Thomas W.P. Friedl, Brigitte Rack, Ellen Heitzer, Fabienne Schochter and Lisa Wiesmüller

Table of contents:

- Supplementary materials and methods
- Supplementary tables
- Supplementary figures
- Supplementary references
- Supplementary Table S7 is available in a separate excel file

Supplementary materials & methods

Cell Lines, Cultivation, and Treatment

MCF-7/182R-6 (RRID:CVCL_W536, provided by American Type Culture Collection, ATCC, Manassas, Virginia, USA) and MDA-MB-436 (RRID:CVCL_0623; provided by University Clinic Ulm, Germany) were cultivated as described in Schochter et al[1]. For drug treatments, the cells were seeded on day 0 and treated with 10µM Olaparib on day 1 for 24h. Cells were harvested for either CyTOF® staining or cell fixation for IF. All human cell lines have been authenticated using STR profiling within the last three years. All experiments were performed with mycoplasma-free cells.

Patient characteristics and sample collection.

For this analysis, 13 patients were selected, of which nine had received genotoxic anticancer therapy, i.e. Anthracycline (Epirubicin, Pegylated Liposomal Doxorubicin (PLD)), Capecitabine, Carboplatin, Cisplatin, Gemcitabine and/or Olaparib after study enrollment, irrespective of prior or subsequent therapeutic agents. For comparison, we included four mBC patients treated with microtubule inhibitory treatment (MTi) (Eribulin, nabPaclitaxel, Vinorelbine). Tumors were classified as hormone receptor positive if they displayed positive estrogen receptor and/or progesterone receptor expression (≥10% for estrogen receptor, ≥10% for progesterone receptor) as determined by immunohistochemistry (Table1). HER2 status was assessed using the HERCEPT test (DAKO, Glostrup, Denmark) on a scale of 0 to 3+, whereby only tumors scoring 3+ or 2+ with detectable HER2 amplification were considered HER2 positive. HER2 amplification was determined using the ZytoMation® ERBB2/CEN 17 Dual Color FISH Probe (Cytovision GmbH, Bremerhaven, Germany). Clinical data were collected from the hospital's internal patient records system.

Blood samples for CTC analysis were collected as scheduled in Table1 prior to the commencement of a new therapy or treatment cycle. During treatments, samples were taken ≥seven days after treatment administration. A total of 22.5ml of whole blood was collected (2x EDTA monovettes 7.5ml, 1x CellSave® tube 7.5ml). Laboratory processing was completed within 12h for EDTA samples and within 96h for CellSave® samples.

PE samples were obtained by thoracentesis from advanced BC patients treated at the Department of Women's Health in Tübingen. PE samples were processed as described in Önder et al[2]. In short, the entire PE sample was centrifuged at 500xg and the resulting cell pellet subjected to red blood cell lysis. The final cell pellet was resuspended in Recovery Cell Culture Freezing Medium (Thermo Fisher, Waltham, MA, USA, 12648010) and cryopreserved long-term in liquid nitrogen.

EDTA blood sample processing and mass cytometry

Two 7.5 ml EDTA blood samples were centrifuged at 2000xg for 10 min and the supernatant transferred to a new tube for a second round of centrifugation at 2000xg for 10 min to prepare plasma samples free of debris. Plasma samples were stored frozen at -80 °C until isolation of cell free DNA (cfDNA). Meanwhile the plasma volume was replaced by phosphate buffered saline (PBS) in the EDTA tubes. The resulting mixture was subjected to Ficoll gradient centrifugation (Ficoll-Plaque® Plus, GE, Cytiva, Marlborough, MA, USA) to isolate peripheral blood mononuclear cells (PBMCs) together with CTCs. Isolated cells were washed in PBS and stored in liquid nitrogen.

The cells were thawed at 4 °C and diluted two-fold by dropwise addition of cold culture medium, namely RPMI 1640 (GIBCO, Invitrogen, Carlsbad, CA) supplemented with 10 % fetal bovine serum (Pan Biotech, Aidenbach, Germany) and 1.0 % Penicillin-Streptomycin-Glutamine (100x) (GIBCO, Invitrogen, Carlsbad, CA), to which Benzonase® Nuklease (Merck Millipore, Billerica, MA, USA) was added to a final concentration of 25 U/ml. The mixture was transferred dropwise into a 10x volume of cold culture medium in 15 ml screw cap tubes (Sarstedt AG & Co. KG, Nümbrecht Germany) followed by gentle mixing and centrifugation at 300xg for 10 min at room temperature (RT). The supernatants were discarded, the pellets resuspended in 10 ml cold medium and centrifuged at 300xg for 10 min at RT. The pellets were resuspended in 1 ml Maxpar® Cell Staining buffer (Standard BioTools Inc., San Francisco, Ca, USA), transferred to Falcon® round-bottom polystyrene test tubes (Corning Inc., Corning, NY, USA) and centrifuged at 300xg for 10 min at RT. Supernatants were discarded and cells incubated for 30 min at RT in a mixture of those metal-conjugated antibodies used to target the selected antigens on the cell surface. Subsequently, the cells were washed with 2 ml Maxpar® Cell Staining buffer and centrifuged for 7 min at 300xg at RT. Supernatants were discarded, pellets resuspended in 1 ml Fixation/Permeabilization working solution (eBioscience™ Fixation/Permeabilization concentrate diluted 1:3 in eBioscience™ Fixation/Permeabilization diluent from Invitrogen/Thermo Fisher Scientific, Waltham, MA, USA), incubated at RT for 1 h and then centrifuged for 7 min at 800xg at RT. Supernatants were discarded and pellets washed twice with 2 ml Permeabilization buffer (eBioscience™ Permeabilization buffer 10x from Invitrogen/Thermo Fisher Scientific, Waltham, MA, USA, diluted 1:10 in Ampuwa plastipur (Fresenius Kabi AG, Bad Homburg vor der Höhe, Germany)) and centrifuged for 7 min at 800xg and RT. Supernatants were discarded and cells incubated for 45 min at RT in a mixture of those metal-conjugated antibodies targeting selected intracellular and intranuclear antigens. The cells were then washed with 2 ml Permeabilization buffer and centrifuged for 7 min at 800xg and RT. This washing step was repeated twice. Then, the cells were incubated in 1.6 % formaldehyde diluted in 1xPBS (GIBCO, Invitrogen, Carlsbad, CA) at 4 °C overnight. The cells were centrifuged for 7 min at 800xg and RT, supernatants were discarded, and the cells stained

with 1 ml Cell-ID™ Intercalator-Ir-125µM (Standard BioTools Inc., San Francisco, Ca, USA) diluted in Maxpar® Fix and Perm buffer (Standard BioTools Inc., San Francisco, CA, USA) and incubated for 1 h at RT. When using cell lines, the dilution factor was 1:3333, for PBMC samples from healthy donors 1:1000 and for patient samples 1:2500. Cells were centrifuged for 7 min at 800xg and RT and washed twice with Maxpar® Cell Staining buffer and once in Milli-Q water. The metal-conjugated antibody-labeled cells were resuspended in 500 µl 10 % DMSO in FBS, transferred to cryotubes and stored at -80 °C.

Finally, labeled cell lines, patient cells and control cells (PBMCs from healthy donors) were analyzed using a Helios™ (a CyTOF® System) mass cytometer, running with software V7.0. The instrument was tuned according to the manufacturer's instructions with tuning solution (Standard BioTools) and measurement of EQ four-element calibration beads (Standard BioTools) served as quality control for sensitivity and recovery. Generated data were normalized and randomized using Helios Software V7. Normalization was performed according to signals elicited by EQ four-element beads. Randomization was applied to values greater than zero by subtracting a uniformly distributed random number between 0 and 1.

Gating strategy for analysis of raw CyTOF files

All data from the different sample types, i. e. patient samples and healthy controls, cell line samples, and PE samples, was analysed as described in the following. Gates were set sequentially.

1. First the calibration beads were excluded: x-axis: Ce140Di upper gate at 100, y-axis: 191Ir lower gate at 100.
2. Singlets gate: x-axis: Ir193, y-axis Ir191. An elliptical gate was set around the main population.
3. Intact singlets: x-axis: Event length upper gate at 30, y-axis: Ir191. A rectangular gate was set around the main population.
4. Live cells: x-axis: Ir191 lower gate at 100, y-axis: cleaved caspase 3 (142Nd) upper gate at 40.
5. $CD45^-$ population: x-axis: Ir191 lower gate at 100, y-axis: CD45 (89Y) upper gate at 2 for patient and control samples, 45 for MCF-7/182R-6 and MDA-MB-436, and 20 for PE samples.
6. $CD45^-/CK^+$ population: x-axis: Ir191 lower gate at 100, y-axis: Pan-CK (162Dy) lower gate at 8 for patient and control samples, and MCF-7/182R-6, and 450 for PE samples.

(For MDA-MB-436 the CD45⁻/CK⁻ population was isolated with the y-axis: Pan-CK (162Dy) upper gate at 8.)

7. $CD45^-/CK^+/EpCAM^+$: x-axis: Ir191 lower gate at 100, y-axis: EpCAM (141Pr) lower gate at 10 for patient and control samples, PE samples and MCF-7/182R-6. (For MDA-MB-436 the $CD45^-/CK^-/EpCAM^-$ population was isolated with the y-axis: EpCAM (141Pr) upper gate at 10.)
8. Biomarkers were all analysed within the $CD45^-/CK^+/EpCAM^+$ population (in case of MDA-MB-436 within the $CD45^-/CK^-/EpCAM^-$ population), x-axis was always Ir191, and the lower gate was set at 100. The biomarkers were all set on the y-axis:
    a. $\gamma H2AX^+$ (147Sm) lower gate at 15 for patient and control samples, PE samples, and MCF-7/182R-6. Lower gate at 40 for MDA-MB-436.
    b. $RAD51^+$ (168Er) lower gate at 55
    c. $RAD51^+$/ $\gamma H2AX^+$: $RAD51^+$ gate within the $\gamma H2AX^+$ population
    d. $53BP1^+$ (173Yb) lower gate at 80
    e. $pRPA32^+$ (176Yb) lower gate at 100
    f. Cyclin A^+ (158Gd) lower gate at 10 for patient and control samples, PE samples, and MDA-MB-436. Lower gate at 15 for MCF-7/182R-6
    g. $Vimentin^+$ (154Sm) lower gate at 20
    h. $ALDH1A3^+$ (110Cd) lower gate at 10
    i. $CD24^-$ (169Tm) upper gate at 6
    j. $CD44^+$ (171Yb) lower gate at 20
    k. $CD44^+/CD24^-$: $CD44^+$ gate within the $CD24^-$ population

For creation of the final figure, CyTOF plots were saved as EMF files without further image editing.

Immunofluorescence microscopy

Cells were seeded on 4-well culture slides (Corning Inc., Corning, NY, USA) and incubated in 1 ml medium containing 10 µM Olaparib for 24 h. Slides were fixed with methanol (Sigma-Aldrich, St. Louis, MO, USA) at −20 °C for 10 min, washed three times in PBS, and blocked with 150 µl PBS with 5 % BSA (Sigma-Aldrich, St-Louis, MO, USA) per chamber for 30 min at

RT. Primary antibodies for RAD51 (anti-Rad51 rabbit H-92, sc-8349 from Santa Cruz Biotechnology, Dallas, TX, USA, 1:500 diluted in PBS with 5% BSA), γH2AX (Anti-phospho Histone H2A.X (Ser139), Alexa Fluor® 488 Conjugate Antibody, 05-636-AF488 from Sigma-Aldrich, St. Louis, MO, USA, 1:150 diluted in PBS with 5% BSA), pRPA32 (Anti-phospho-RPA32 (Ser33), A300-246A from Bethyl, Fortis Life Sciences, Waltham, MA, USA, 1:500 in PBS with 5% BSA), 53BP1 (p53BP1 Polyclonal Antibody, AbBy Fluor® 555 Conjugated, BS-2740R-BF555 from Bioss Inc., Woburn, MA, USA, 1:150 in PBS with 5% BSA), Cyclin A2 (Recombinant Alexa Fluor® 647 Anti-Cyclin A2 antibody [Y193], ab211805 from Abcam, Cambridge, UK. 1:150 in PBS with 5% BSA), EpCAM (Alexa Fluor® 488 anti-human CD326 (EpCAM) Antibody, 324209 from BioLegend, San Diego, CA, USA, 1:150 in PBS with 5% BSA), panCK (Pan-Keratin (C11) Mouse mAb (Alexa Fluor® 555 Conjugate), 3478 from Cell Signaling technology, Denvers, MA. USA, 1:150 in PBS with 5% BSA) and Vimentin (Vimentin (D21H3) XP® Rabbit mAb (Alexa Fluor® 647 Conjugate), 9856 from Cell Signaling technology, Danvers, MA. USA, 1:150 in PBS with 5% BSA) were added and incubated at 4 °C overnight. Secondary anti-rabbit (Alexa Fluor 594 goat anti rabbit IgG H+L, A11037 from Invitrogen/Thermo Fisher Scientific, Waltham, MA, USA, 1:500 diluted in 0.1% PBS-Tween) and anti-rabbit antibodies (Goat anti-Rabbit IgG (H+L) Cross-Adsorbed Secondary Antibody, Alexa Fluor™ 488, A11008 from Invitrogen/Thermo Fisher Scientific, 1:1000 diluted in 0.1 % PBS-Tween) were added and incubated at 37 °C for 45 min protected from light. Slides were washed three times with 0.1 % PBS-Tween and one time with PBS. Coverslips were mounted on slides using VECTASHIELD® Antifade Mounting Medium with DAPI (Vector Laboratories, Newark, CA, USA) and stored at 4 °C. The slides were subjected to high content imaging with an Axio Observer 7 microscope (Carl Zeiss AG, Oberkochen, Germany), objective Plan-Apochromat 100x/1,4 Oil DIC M27, and automated analysis with Zeiss ZEN 3.1 (blue edition) software (Carl Zeiss AG, Oberkochen, Germany). Cells were scored as positive for EpCAM, panCK, Vimentin and Cyclin A if the mean intensity of the cell was more than 300. Cells were scored positive for RAD51, γH2AX and 53BP1 if more or equal to five foci were detected. They were scored positive for pRPA32 if more or equal to 30 foci were detected.

HRD scoring of genomic DNA from PE samples

The HRD score was determined by clinical diagnostics as part of a molecular tumor board and extracted from the provided sequencing report. In short, coding genomic target regions, the neighbouring intron regions and selected individual introns (necessary for the detection of certain fusions) were enriched using Agilent's in-solution technology (SureSelectXT) and then sequenced on an Illumina system (NextSeq500) as 2x75 bp paired-end reads. The assessment of HRD score is based on the sum of copy number changes such as loss of

heterozygosity, telomeric allelic imbalance and LSTs in the tumor sample as described in Telli et al[3]. The cut-off value was set to 42.

Statistical analyses

Graphic presentation of data was performed using GraphPad Prism 9 software (La Jolla, CA, USA). For statistically significant differences in two-sample cases obtained via IF or CyTOF® either Wilcoxon-matched pair signed-ranks test (non-parametric related samples) or two-tailed Mann-Whitney U test (non-parametric independent samples) were used. In k-sample cases obtained via CyTOF® two-tailed Mann-Whitney U test was used in case of statistical significance being reached with Kruskal-Wallis H-test. For correlation analyses two-tailed Spearman correlation with 95% confidence interval was used. *: $P<0.05$, **: $P<0.01$, ***: $P<0.001$, ****: $P<0.0001$.

Supplementary tables

Supplementary Table 1. Summary of mBC patient characteristics together with various CTC/cell subpopulations from longitudinally collected blood samples

Pat. No.	primary tumor subtype	metastasis subtype	sample date (day.month.year)	treatment status	Line of treatment in the metastatic setting	CTC number * (CellSearch®)	CTC number * (CyTOF®) CD45^{-}/panCK^{+}/EpCAM^{+}	cell number * (CyTOF®) CD45^{-}/EpCAM^{+}	cell number * (CyTOF®) CD45^{-}/panCK^{+}	cell number * (CyTOF®) CD45^{-}/panCK^{-}/EpCAM^{+}	cell number * (CyTOF®) CD45^{+}/panCK^{+}	cell number * (CyTOF®) CD45^{-}/Vimentin^{+}	cell number * (CyTOF®) CD45^{-}/Vimentin^{+}/panCK^{+}
# 07	TNBC	TNBC	26.01.2018	NT		17	2	43.5	4.5	41.5	22	50987.5	2
			22.02.2018	GT	5.	9	1.5	30.5	5.5	29	79	55493.5	3
			06.04.2018	GT		7	1	80	3.5	79	37	30771	0.5
# 11	HR+/HER2+	HR+/HER2+	17.10.2017	NT		89	1.5	61.5	4.5	60	124	19818	1.5
			07.11.2017	GT	4.	2	0	29	3	29	21.5	25985.5	2
			19.12.2017	GT		16	0	16	6	16	31.5	33547	3.5
			20.02.2018	GT		39	5	62.5	13.5	57.5	79.5	91431	4
# 20	TNBC	HER2+	05.02.2018	NT		0	0	15.5	8.5	15.5	142	43728.5	1
			26.02.2018	MTi	1.	0	0.5	5.5	9	5	152	8637.5	0.5
			19.03.2018	MTi		0	372.5	417	375.5	44.5	139	45614	41.5
			14.05.2018	MTi		0	1	32.5	4.5	31.5	143.5	97018.5	2.5
			04.06.2018	MTi		4	146.5	372.5	149	226	98.5	18591	2
			15.06.2018	MTi		186	6	11	8	5	30.5	9730	0
			25.07.2018	NT		5	0	3.5	13	3.5	226	5096	0
			04.09.2018	NT		0	1	93.5	2	92.5	21.5	68602	0.5
			16.10.2018	MTi	2.	2	7.5	27	8.5	19	27	12938.5	0
			07.03.2019	GT	4.	0	1	109.5	5	108	45	95253	1
# 46	HR+/HER2-	HR+/HER2-	14.03.2019	NT		5	59	61.5	64.5	2.5	171	50	5
			16.04.2019	NT		5	2	194.5	17	192.5	2495	39152	2
			25.02.2020	GT	4.		4	18.5	10	14.5	79	1065	0.5
			03.03.2020	GT			21.5	28.5	29.5	7	55.5	3948	1.5
# 49	HR+/HER2-	HR+/HER2-	16.04.2019	NT		0	0.67	91.3	11.3	136	534	11845.4	0.3
			07.05.2019	MTi	3.	0	3.5	20.5	22.5	17	801	17768	0.5
# 55	HR+/HER2-	TNBC	25.06.2019	NT		8	13.2	336.5	26.5	326.5	706.5	29829.5	6
			12.11.2019	GT	2.		2.5	217	17.5	214.5	357.5	6970.5	4.5
			12.02.2020	GT	3.		5	191.5	22	186.5	67.5	4270.5	3.5
			16.03.2020	GT			5	222	18.5	217	305	6308.5	4.5
# 57	HR+/HER2-	TNBC	01.07.2019	NT		0	4	546	19.5	543	802	52872	11
			12.07.2019	NT		0	5.5	423.5	18	418	715	30867	7.5
			16.08.2019	GT	2.	0	3.5	386.5	21	383	642	27304	8
			21.02.2020	GT			6.5	459	64.5	452.5	845.5	16607.5	33
# 61	TNBC	liver: HR+<br>skin: TNBC	05.11.2019	NT		210	2	984.5	4	975.5	966	18042.5	15
			26.11.2019	GT	3.		25	1036.5	47	1011.5	428	8187.5	16
			17.12.2019	GT			1.5	828.5	6	827	91.5	456.5	0
			14.02.2020	GT	4.		2	602.5	7.5	600.5	262.5	1720.5	3.5
			27.03.2020	GT			7.5	759.5	19	752	220	8605	9
			21.08.2020	MTi	5.	16	2	710.5	17.5	708.5	341.5	53160	5
			11.09.2020	MTi			13	1671.5	45.5	1658.5	1179.5	20737.5	26
			02.10.2020	MTi		7	9	984.5	29	975.5	966	18042.5	15
# 62	TNBC	TNBC	08.04.2020	NT		38	8	1120	21	1114	371	23893	13.5
			14.07.2020	GT	1.		1	184.5	7.5	183.5	564	10509.5	3
			24.08.2020	NT		7	3.5	293	8.5	289.5	296.5	4271.5	7
# 70	TNBC	TNBC	22.12.2020	NT		267	168.7	310.7	318.7	142	3012	73806	107.3
#79	HR+/HER2-	HR+/HER2-	07.09.2021	NT		0	0.5	12	2	11.5	100	5780	0.5
			07.10.2021	MTi	3.	0	0	8	2.5	9.5	59	27332	0
			04.11.2021	MTi		1	10.5	34	21	23.5	264.5	21312	2.5
# 85	TNBC	TNBC	17.12.2021	NT		7	4.5	1479.5	13.5	1475	630.5	29494	2.5
# 106	HR+/HER2-	TNBC	23.02.2023	NT		81	11.5	578.5	26	567	473	40768	4.5
			23.03.2023	GT	1.		4	713.5	12.5	709.5	407.5	29408.5	2.5
			24.05.2023	NT		8	2	712.5	8.5	710.5	660.5	34669	2
			14.06.2023	GT	2.	3	0.5	60	1.5	59.5	13	113	0.5
			20.03.2024	GT	3.	62	12	60	14.5	48	165.5	54769	1

* CTC/cell numbers are given per 7.5 ml blood, whereby a total volume of 7.5 ml blood was analyzed via CellSearch® and of at least 15 ml blood via CyTOF®.
GT, blood draw during genotoxic treatment (lilac shading) (Doxorubicin, Epirubicin, Carboplatin+nabPaclitaxel, Carboplatin+Gemcitabin, Carboplatin, Olaparib, Capecitabine+Lapatinib, Sacituzumab-Govitecan);
HER2+, human epidermal growth factor receptor positive;
HR+, hormone receptor positive;
MTi, blood draw during treatment with microtubule inhibitor (blue shading) (Vinorelbin+Trastuzumab+Pertuzumab, Eribulin, Paclitaxel+Bevacizumab, nabPaclitaxel, TDM-1);
NT, no treatment;
Pat. No., patient number;
TNBC, triple-negative breast cancer;

Supplementary Table 2. CyTOF Antibodies

Antibody	Source
Anti-Human CD45 (HI30)-89Y	Standard BioTools Inc., San Francisco, Ca, USA
Anti-Human CD326/EpCAM (9C4)-141Pr	Standard BioTools Inc., San Francisco, Ca, USA
Anti-Cleaved Caspase 3 (D3E9)-142Nd	Standard BioTools Inc., San Francisco, Ca, USA
Anti-Human Pan-Keratin (C11)-162Dy	Standard BioTools Inc., San Francisco, Ca, USA
Anti-Vimentin (D21H3)-154Sm	Standard BioTools Inc., San Francisco, Ca, USA
Anti-p-Histone H2A.X [Ser139] (JBW301)-147Sm	Standard BioTools Inc., San Francisco, Ca, USA
Anti-Human CyclinA (BF683)-158Gd	Standard BioTools Inc., San Francisco, Ca, USA
Anti-Human/Mouse CD44 (IM7)-171Yb	Standard BioTools Inc., San Francisco, Ca, USA
Anti-Human CD24 (ML5)-169Tm	Standard BioTools Inc., San Francisco, Ca, USA
Phospho-RPA32 (Ser33) - 176Yb	Standard BioTools Inc., San Francisco, Ca, USA
53BP1 Antibody #4937 – 173Yb	Cell Signaling Technology, Danvers, MA, USA
Rad51 Antibody (F-11) – 168Er	Santa Cruz Biotechnology, Dallas, TX, USA
ALDH1A3 Monoclonal Antibody (GT926) – Cd110	Invitrogen/Thermo Fisher Scientific, Waltham, MA, USA
Maxpar® X8 Antibody Labeling Kit, 173Yb	Standard BioTools Inc., San Francisco, Ca, USA
Maxpar® X8 Antibody Labeling Kit, 168Er	Standard BioTools Inc., San Francisco, Ca, USA
Maxpar® MCP9 Antibody Labeling Kit, 114Cd	Standard BioTools Inc., San Francisco, Ca, USA

Supplementary Table 3. CyTOF Antibody Panel

**Cell lineage marker**	
CD45	Pan leukocyte
Pan-cytokeratin	Epithelial
EpCAM	Epithelial
**Epithelial-mesenchymal transition**	
Vimentin	Mesenchymal
**Stemness**	
CD44	Stemness
CD24	Stemness
ALDH1A3	Stemness
**DNA damage**	
γH2AX	DNA damage repair
RAD51	Homologous recombination
53BP1	modulation of end resection/<br>marker of DNA ends and non-homologous end joining
pRPA32	DNA replication/DNA damage repair
**Cell Cycle and Apoptosis**	
Cyclin A	S phase
Cleaved caspase 3	Apoptosis

Supplementary Table 4. Summary of mBC patient characteristics together with cell (CD45$^-$/EpCAM$^+$) features from longitudinally collected blood samples

Pat. No.	primary tumor subtype	metastasis subtype	sample date (day.month.year)	treatment status	Line of treatment in the metastatic setting	CTC number * (CellSearch®)	cell number * (CyTOF®) CD45$^-$/EpCAM$^+$	HRD § (ctDNA®)	HRD $ (CyTOF®)	γH2AX$^+$ (%)	RAD51$^+$ (%)	RAD51$^+$/γH2AX$^+$ (%)	53BP1$^+$ (%)	Cyclin A$^+$ (%)	CD44$^+$/CD24$^-$ (%)	ALDH1A3$^+$ (%)	Vimentin$^+$ (%)	pRPA32$^+$ (%)
# 07	TNBC	TNBC	26.01.2018	NT		17	43.5		YES	20.7	1.15	0.0	2.3	2.3	0.0	2.3	35.6	0.0
			22.02.2018	GT	5.	9	30.5		YES	29.5	1.6	0.0	4.9	3.3	2.3	3.3	27.9	0.0
			06.04.2018	GT		7	80		YES	12.5	0.0	0.0	0.6	0.0	0.0	1.3	46.0	0.0
# 11	HR+/HER2+	HR+/HER2+	17.10.2017	NT		89	61.5		n.a	4.1	1.6	20	9.8	1.6	0.0	4.	54.9	0.0
			07.11.2017	GT	4.	2	29		n.a	3.5	0.0	0.0	3.5	0.0	4.4	3.5	32.8	0.0
			19.12.2017	GT		16	16		YES	25.0	0.0	0.0	6.3	0.0	8.7	0.0	34.4	0.0
			20.02.2018	GT		39	62.5		YES	16.8	0.0	0.0	5.6	0.0	7.6	2.4	32.8	0.0
# 20	TNBC	HER2+	05.02.2018	NT		0	15.5	NO	YES	37.9	0.0	0.0	0.0	3.5	5.0	0.0	41.4	0.0
			26.02.2018	MTi	1.	0	5.5		n.a	9.1	0.0	0.0	0.0	0.0	25.0	9.1	89.4	0.0
			19.03.2018	MTi		0	417		NO	12.0	3.8	6.0	32.9	2.0	4.1	0.4	12.7	27.5
			14.05.2018	MTi		0	47.5		YES	15.4	0.0	0.0	0.0	0.0	4.2	1.5	44.6	0.0
			04.06.2018	MTi		4	372.5		n.a	8.2	0.0	0.0	23.8	2.0	2.0	0.3	6.6	0.7
			15.06.2018	MTi		186	11		YES	18.2	0.0	0.0	9.1	0.0	0.0	0.0	63.6	4.6
			25.07.2018	NT		5	3.5		n.a	0.0	0.0	0.0	0.0	28.6	33.3	14.3	71.4	0.0
			04.09.2018	NT		0	93.5		YES	11.2	0.0	0.0	0.0	0.0	0.0	1.1	87.8	0.0
			16.10.2018	MTi	2.	2	27	NO	YES	38.9	0.0	0.0	7.4	3.7	4.1	1.8	11.1	0.0
			07.03.2019	GT	4.	0	109.5		YES	11.9	0.5	0.0	0.0	1.4	0.0	2.3	16.4	0.0
# 46	HR+/HER2-	HR+/HER2-	14.03.2019	NT		5	61.5	NO	NO	19.9	4.1	4.6	68.3	7.3	0.0	1.6	16.8	14.6
			16.04.2019	NT		5	194.5	YES	NO	20.3	2.1	1.3	0.5	1.3	2.0	2.8	10.3	0.0
			25.02.2020	GT	4.		18.5	YES	YES	27.0	0.0	0.0	10.8	0.0	25.0	0.0	13.5	0.0
			03.03.2020	GT			28.5	YES	YES	26.3	1.8	0.0	43.9	1.8	70.5	0.0	5.3	3.5
# 49	HR+/HER2-	HR+/HER2-	16.04.2019	NT		0	91.3		n.a	8.8	0.0	0.0	0.4	0.7	0.0	15.3	1.8	0.0
			07.05.2019	MTi	3.	0	20.5		n.a	2.4	0.0	0.0	4.9	0.0	0.0	19.5	36.8	0.0
# 55	HR+/HER2-	TNBC	25.06.2019	NT		8	336.5	NO	NO	40.4	2.7	1.8	0.7	4.2	2.2	4.6	33.3	0.0
			12.11.2019	GT	2.		217	NO	NO	65.0	1.2	1.1	0.5	4.2	0.7	3.0	35.0	0.5
			12.02.2020	GT	3.		191.5	NO	NO	84.6	1.0	1.2	2.1	4.4	1.1	2.6	16.2	0.3
			16.03.2020	GT			222	NO	NO	59.9	0.7	1.1	0.7	5.2	2.6	1.6	44.8	0.0
# 57	HR+/HER2-	TNBC	01.07.2019	NT		0	546		YES	53.2	0.6	0.3	0.1	5.6	0.7	1.5	49.5	0.0
			12.07.2019	NT		0	423.5		NO	20.4	1.8	1.2	0.0	4.4	4.0	0.9	23.3	0.0
			16.08.2019	GT	2.	0	386.5		YES	71.4	0.1	0.2	0.7	4.5	0.9	1.9	62.4	0.0
			21.02.2020	GT			459		YES	69.1	0.2	0.2	0.9	3.7	11.1	3.1	53.8	0.9
# 61	TNBC	liver: HR+ skin: TNBC	05.11.2019	NT		210	984.5		YES	69.2	0.0	0.0	0.2	2.2	0.1	5.5	27.3	0.0
			26.11.2019	GT	3.		1036.5		YES	52.1	0.8	0.8	1.4	3.8	0.9	2.9	63.7	1.5
			17.12.2019	GT			828.5		YES	82.3	0.6	0.7	0.6	1.5	0.8	4.5	5.61	0.0
			14.02.2020	GT	4.		602.5		YES	83.7	0.2	0.2	0.2	2.0	0.4	8.2	19.6	0.0
			27.03.2020	GT			759.5		NO	36.1	1.0	1.1	0.0	2.6	0.8	5.6	56.9	0.0
			21.08.2020	MTi	5.	16	710.5		YES	30.3	0.5	0.7	0.0	0.8	0.9	3.4	47.6	0.0
			11.09.2020	MTi			1671.5		YES	55.0	0.5	0.5	0.5	1.6	1.5	2.9	59.1	0.2
			02.10.2020	MTi		7	984.5		YES	40.1	0.4	0.8	0.3	2.3	1.3	3.2	64.7	0.0
# 62	TNBC	TNBC	08.04.2020	NT		38	1120	Borderline	YES	30.3	0.0	0.0	0.1	0.4	0.5	2.1	63.8	0.2
			14.07.2020	GT	1.		184.5		YES	59.6	0.0	0.0	0.3	0.5	4.0	6.8	56.6	0.5
			24.08.2020	NT		7	293		YES	17.4	0.2	0.0	0.7	3.4	2.5	7.5	59.2	0.0
# 70	TNBC	TNBC	22.12.2020	NT		267	310.7	Borderline	NO	42.7	4.9	10.6	1.3	18.0	0.4	5.8	25.5	1.3
#79	HR+/HER2-	HR+/HER2-	07.09.2021	NT		0	12	NO	YES	25.0	4.2	0.0	0.0	0.0	0.0	8.3	8.3	0.0
			07.10.2021	MTi	3.	0	8		n.a	0.0	0.0	0.0	0.0	0.0	0.0	26.3	0.0	0.0
			04.11.2021	MTi		1	34	NO	YES	14.7	1.5	0.0	14.7	1.5	0.0	17.6	11.8	5.9
# 85	TNBC	TNBC	17.12.2021	NT		7	1479.5		YES	31.2	0.5	0.7	0.1	0.5	0.1	2.6	29.6	0.0
# 106	HR+/HER2-	TNBC	23.02.2023	NT		81	578.5	YES	YES	25.0	0.2	0.0	0.2	3.4	0.8	6.2	23.0	0.0
			23.03.2023	GT	1.		713.5		YES	46.0	0.1	0.2	0.1	2.6	0.0	4.1	17.7	0.0
			24.05.2023	NT		8	712.5	Borderline	YES	47.6	0.1	0.2	0.2	3.7	0.5	3.4	43.9	0.0
			14.06.2023	GT	2.	3	60		YES	38.3	2.5	0.0	5.0	4.2	1.8	25.0	9.2	0.0
			20.03.2024	GT	3.	62	60		YES	32.5	0.8	0.0	18.3	2.5	3.8	8.3	15.0	0.0

* CTC/cell numbers are given per 7.5 ml blood, whereby a total volume of 7.5 ml blood was analyzed via CellSearch® and of at least 15 ml blood via CyTOF®.

§ HRD (ctDNA) metric is based on the detection of large genomic alterations (LGAs) in ctDNA, defined as breaks larger than 3Mb that are within 10Mb of each other. >20 LGAs identifies HRD (YES), 15-19 Borderline HRD (Borderline), 0-14 no HRD (NO).

$ HRD (CyTOF®) metric is based on the detection of γH2AX$^+$ CTCs with RAD51$^+$/γH2AX$^+$ <1.0% (YES) as compared to γH2AX+ CTCs with RAD51$^+$/γH2AX$^+$ ≥1.0% (NO). Concordance between HRD detection via ctDNA® (YES/Borderline) and CyTOF® (YES) is marked by green shading, discordance (NO) by red shading.

GT, blood draw during genotoxic treatment (lilac shading) (Doxorubicin, Epirubicin, Carboplatin+nabPaclitaxel, Carboplatin+Gemcitabin, Carboplatin, Olaparib, Capecitabine+Lapatinib, Sacituzumab-Govitecan);

HER2+, human epidermal growth factor receptor positive;

HR+, hormone receptor positive;

MTi, blood draw during treatment with microtubule inhibitor (blue shading) (Vinorelbin+Trastuzumab+Pertuzumab, Eribulin, Paclitaxel+Bevacizumab, nabPaclitaxel, TDM-1);

n.a., not applicable regarding HRD (CyTOF®) categorization, if total number of CTCs<3 or RAD51$^+$/γH2AX$^+$<1% but γH2AX$^+$ <10%;

NT, no treatment;

Pat. No., patient number;

TNBC, triple-negative breast cancer;

Supplementary Table 5. Summary of mBC patient characteristics together with cell ($CD45^{-}/panCK^{+}$) features from longitudinally collected blood samples

Pat. No.	primary tumor subtype	metastasis subtype	sample date (day.month.year)	treatment status	Line of treatment in the metastatic setting	CTC number * (CellSearch®)	cell number * (CyTOF®) $CD45^{-}/panCK^{+}$	HRD § (ctDNA®)	HRD $ (CyTOF®)	$\gamma H2AX^{+}$ (%)	$RAD51^{+}$ (%)	$RAD51^{+}/\gamma H2AX^{+}$ (%)	$53BP1^{+}$ (%)	Cyclin A^{+} (%)	$CD44^{+}/CD24^{-}$ (%)	$ALDH1A3^{+}$ (%)	$Vimentin^{+}$ (%)	$pRPA32^{+}$ (%)
# 07	TNBC	TNBC	26.01.2018	NT		17	4.5		YES	11.1	0.0	0.0	11.1	0.0	0.0	0.0	44.4	0.0
			22.02.2018	GT	5.	9	5.5		YES	45.5	0.0	0.0	9.1	0.0	0.0	0.0	54.5	0.0
			06.04.2018	GT		7	3.5		YES	14.3	0.0	0.0	14.3	0.0	20.0	0.0	14.3	0.0
# 11	HR+/HER2+	HR+/HER2+	17.10.2017	NT		89	4.5		n.a.	0.0	11.1	0.0	33.3	0.0	0.0	22.2	54.9	0.0
			07.11.2017	GT	4.	2	3		YES	16.7	0.0	0.0	50.0	16.7	0.0	0.0	66.7	0.0
			19.12.2017	GT		16	6		YES	41.7	0.0	0.0	8.3	0.0	27.3	0.0	58.3	0.0
			20.02.2018	GT		39	13.5		YES	14.8	0.0	0.0	18.5	0.0	8.0	3.7	29.6	0.0
# 20	TNBC	HER2+	05.02.2018	NT		0	8.5	NO	n.a.	5.9	0.0	0.0	0.0	0.0	0.0	5.9	11.8	0.0
			26.02.2018	MTi	1.	0	9		n.a.	0.0	0.0	0.0	0.0	0.0	33.3	5.6	5.6	0.0
			19.03.2018	MTi		0	375.5		n.a.	9.6	4.3	8.3	36.4	2.1	3.3	0.5	11.1	30.5
			14.05.2018	MTi		0	4.5		n.a.	0.0	0.0	0.0	0.0	0.0	25.0	0.0	55.6	0.0
			04.06.2018	MTi		4	149		n.a.	5.0	0.0	0.0	59.4	4.4	0.0	0.3	1.3	1.7
			15.06.2018	MTi		186	8		YES	10.5	0.0	0.0	15.8	0.0	0.0	0.0	0.0	5.3
			25.07.2018	NT		5	13		n.a.	3.9	0.0	0.0	0.0	0.0	0.0	0.0	0.0	0.0
			04.09.2018	NT		0	2		YES	25.0	0.0	0.0	0.0	0.0	0.0	0.0	25.0	0.0
			16.10.2018	MTi	2.	2	8.5	NO	n.a.	5.9	0.0	0.0	23.5	0.0	0.0	5.9	0.0	0.0
			07.03.2019	GT	4.	0	5		YES	10.0	0.0	0.0	0.0	0.0	0.0	0.0	20.0	0.0
# 46	HR+/HER2-	HR+/HER2-	14.03.2019	NT		5	64.5	NO	NO	16.3	3.9	4.8	65.9	7.0	0.0	2.3	7.8	14.0
			16.04.2019	NT		5	17	YES	NO	14.7	38.2	40.0	26.5	32.4	18.2	32.4	11.8	14.7
			25.02.2020	GT	4.		10	YES	YES	15.0	0.0	0.0	25.0	5.0	46.7	5.0	5.0	0.0
			03.03.2020	GT			29.5	YES	YES	23.7	1.7	0.0	44.1	1.7	68.9	3.4	5.1	3.4
# 49	HR+/HER2-	HR+/HER2-	16.04.2019	NT		0	11.3		n.a.	5.9	2.9	50.0	2.9	0.0	0.0	41.2	2.9	0.0
			07.05.2019	MTi	3.	0	22.5		n.a.	0.0	0.0	0.0	6.7	0.0	0.0	51.1	2.2	0.0
# 55	HR+/HER2-	TNBC	25.06.2019	NT		8	26.5	NO	NO	24.5	17.0	15.4	15.1	15.1	25.0	34.0	22.6	7.55
			12.11.2019	GT	2.		17.5	NO	NO	57.1	5.7	10.0	8.6	8.6	3.6	20.0	25.7	2.9
			12.02.2020	GT	3.		22	NO	NO	75.0	20.5	27.3	20.5	25.0	5.88	22.7	15.9	18.2
			16.03.2020	GT			18.5	NO	NO	48.6	8.1	11.1	8.1	10.8	9.68	10.8	24.3	2.7
# 57	HR+/HER2-	TNBC	01.07.2019	NT		0	19.5		YES	35.9	7.7	0.0	5.1	17.9	6.9	20.5	56.4	5.1
			12.07.2019	NT		0	18		NO	16.7	5.6	16.7	2.8	5.6	0.0	27.8	41.7	0.0
			16.08.2019	GT	2.	0	21		NO	31.0	1.4	7.7	7.1	7.1	6.9	31.0	45.2	2.4
			21.02.2020	GT			64.5		YES	69.8	0.8	0.0	3.9	7.8	8.3	5.4	51.2	0.8
# 61	TNBC	liver: HR+ skin: TNBC	05.11.2019	NT		210	4		YES	62.5	0.0	0.0	12.5	25.0	0.0	0.0	0.0	0.0
			26.11.2019	GT	3.		47		NO	48.9	11.7	6.5	30.9	9.6	0.0	10.6	34.0	26.6
			17.12.2019	GT			6		YES	75.0	8.3	0.0	8.3	0.0	0.0	0.0	0.0	0.0
			14.02.2020	GT	4.		7.5		YES	46.7	0.0	0.0	26.7	13.3	27.3	0.0	46.7	0.0
			27.03.2020	GT			19		YES	26.3	2.3	0.0	0.0	13.2	0.0	7.9	47.4	0.0
			21.08.2020	MTi	5.	16	17.5		YES	17.1	0.0	0.0	0.0	0.0	0.0	28.6	28.6	0.0
			11.09.2020	MTi			45.5		NO	45.1	3.3	2.4	2.2	4.4	5.5	11.0	57.1	1.1
			02.10.2020	MTi		7	29		YES	19.0	5.2	0.0	8.6	3.5	7.3	13.8	51.7	0.0
# 62	TNBC	TNBC	08.04.2020	NT		38	21	Borderline	YES	33.3	0.0	0.0	4.8	4.8	5.0	4.8	64.3	7.1
			14.07.2020	GT	1.		7.5		YES	53.3	0.0	0.0	0.0	6.7	25.0	20.0	40.0	0.0
			24.08.2020	NT		7	8.5		n.a.	5.9	5.9	0.0	5.9	17.6	0.0	5.9	82.4	0.0
# 70	TNBC	TNBC	22.12.2020	NT		267	318.7	Borderline	NO	45.4	10.9	20.3	2.7	28.9	1.1	5.4	33.7	3.1
#79	HR+/HER2-	HR+/HER2-	07.09.2021	NT		0	2	NO	YES	25.0	0.0	0.0	0.0	25.0	0.0	25.0	25.0	0.0
			07.10.2021	MTi	3.	0	2.5		n.a.	0.0	0.0	0.0	0.0	0.0	0.0	0.0	0.0	0.0
			04.11.2021	MTi		1	21	NO	n.a.	7.1	0,.0	0.0	23.8	0.0	0.0	31.0	11.9	9.5
# 85	TNBC	TNBC	17.12.2021	NT		7	13.5		YES	11.1	0.0	0.0	0.0	7.4	5.9	22.2	18.5	0.0
# 106	HR+/HER2-	TNBC	23.02.2023	NT		81	26	YES	n.a.	9.6	13.5	20.0	11.5	25.0	0.0	23.1	17.2	5.8
			23.03.2023	GT	1.		12.5		YES	12.0	8.0	0.0	8.0	8.0	9.1	36.0	20.0	0.0
			24.05.2023	NT		8	8.5	Borderline	YES	11.8	41.2	0.0	17.6	41.2	0.0	58.8	23.5	11.8
			14.06.2023	GT	2.	3	1.5		n.a.	0.0	0.0	0.0	0.0	0.0	0.0	0.0	33.3	0.0
			20.03.2024	GT	3.	62	14.5		YES	24.1	3.5	0.0	55.2	3.5	0.0	3.5	6.9	0.0

* CTC/cell numbers are given per 7.5 ml blood, whereby a total volume of 7.5 ml blood was analyzed via CellSearch® and of at least 15 ml blood via CyTOF®.
§ HRD (ctDNA) metric is based on the detection of large genomic alterations (LGAs) in ctDNA, defined as breaks larger than 3Mb that are within 10Mb of each other. >20 LGAs identifies HRD (YES), 15-19 Borderline HRD (Borderline), 0-14 no HRD (NO).
$ HRD (CyTOF®) metric is based on the detection of $\gamma H2AX^{+}$ CTCs with $RAD51^{+}/\gamma H2AX^{+}$ <1.0% (YES) as compared to γH2AX+ CTCs with $RAD51^{+}/\gamma H2AX^{+}$ ≥1.0% (NO). Concordance between HRD detection via ctDNA® (YES/Borderline) and CyTOF® (YES) is marked by green shading, discordance (NO) by red shading.
GT, blood draw during genotoxic treatment (lilac shading) (Doxorubicin, Epirubicin, Carboplatin+nabPaclitaxel, Carboplatin+Gemcitabin, Carboplatin, Olaparib, Capecitabine+Lapatinib, Sacituzumab-Govitecan);
HER2+, human epidermal growth factor receptor positive;
HR+, hormone receptor positive;
MTi, blood draw during treatment with microtubule inhibitor (blue shading) (Vinorelbin+Trastuzumab+Pertuzumab, Eribulin, Paclitaxel+Bevacizumab, nabPaclitaxel, TDM-1);
n.a., not applicable regarding HRD (CyTOF®) categorization, if total number of CTCs<3 or $RAD51^{+}/\gamma H2AX^{+}$<1% but $\gamma H2AX^{+}$ <10%;
NT, no treatment;
Pat. No., patient number;
TNBC, triple-negative breast cancer;

Supplementary Table 6. Summary of pleural effusion sample analysis

Pat. No.	pleura sample date (day.month.year):	treatment status	HRD score[§] (genomic)	tumor sample used for determination of HRD score	reported genomic changes of HRD genes	HRD[$] (CyTOF®)	γH2AX$^+$ (%)	RAD51$^+$ (%)	RAD51$^+$/γH2AX$^+$ (%)	53BP1$^+$ (%)	Cyclin A$^+$ (%)	CD44$^+$/CD24$^-$ (%)	ALDH1A3$^+$ (%)	Vimentin$^+$ (%)
PL-TUE #01	17.07.2023	NT	5 (HRD ≥ 42)	tumor sample from pleural puncture	*ERCC4* c.2395C>T, p.Arg799Trp	NO	61.4	4.8	3.5	99.5	5.3	95.7	7.9	54.5
PL-TUE #02	15.10.2021	GT	28 (HRD ≥ 42)	invasive mammary carcinoma (G3) from 2019	*BRCA1* & *2* DEL (het), *BRCA2* AMP (5 copies), *RAD51D* DEL (het), *CDK12* DEL (het)	NO	31.7	2.9	1.4	98.3	5.3	52.0	0.9	1.5
PL-TUE #03	15.01.2024	GT	60 (HRD ≥ 42)	osseous metastases (Vertebra thoracica VII) from 2020	*CHEK2* c.1277del, p.Pro426LeufsTer11; AMP (4 copies)	YES	35.7	0.0	0.0	85.7	0.0	66.7	0.0	21.4

[§] HRD score (genomic) metric is based on the sum of copy number changes such as loss of heterozygosity, telomeric allelic imbalance and large-scale transitions in the tumor sample as described in Telli et al[2].
[$] HRD (CyTOF®) metric is based on the detection of γH2AX$^+$ CTCs without RAD51-positivity (YES) as compared to γH2AX$^+$ CTCs with RAD51-positivity (NO).
GT: pleura sample punctated during genotoxic treatment (lilac shading) (Carboplatin+Olaparib, Sacituzumab-Govitecan)
Pat. No., patient number

Supplementary figures

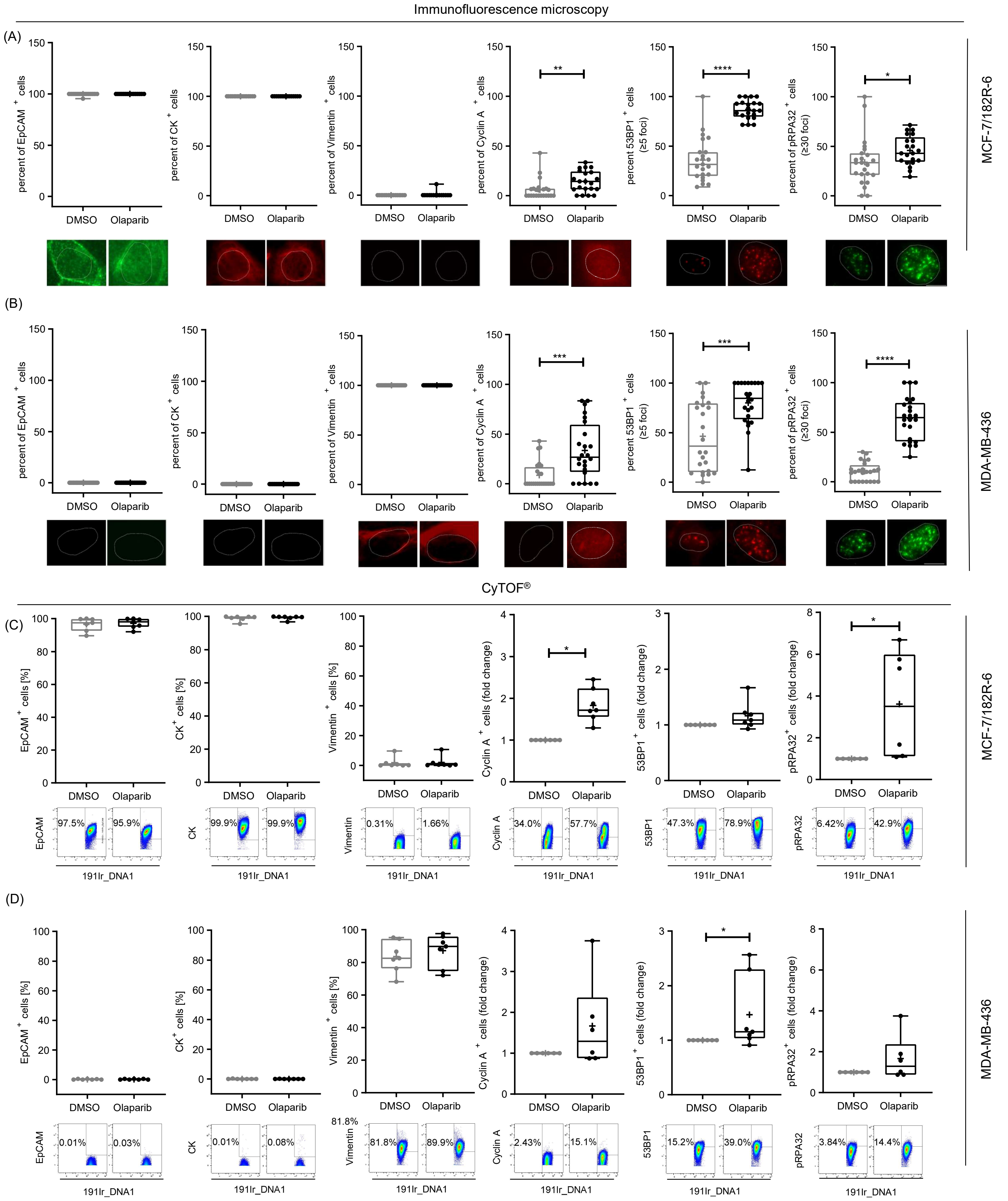

Supplementary Figure 1: Immunofluorescence microscopy was performed with MCF-7/182R-6 (A) and MDA-MB-436 (B) cells after DMSO and Olaparib (10 µM) treatment for 24 h and staining for EpCAM, CK, Vimentin, Cyclin A, 53BP1 and pRPA32. The fractions of EpCAM⁺, CK⁺, Vimentin⁺, Cyclin A⁺, 53BP1⁺ and pRPA32⁺ cells are given in percent. Between 10-15 images per experiment from two individual experiments were analyzed, respectively. Boxes, intraquartile range; horizontal lines, median; cross, mean; whiskers, min to max. Significances were calculated by Mann-Whitney U test using GraphPad Prism 9. *: P<0.05, **: P<0.01, ***: P<0.001, ****: P<0.0001. CyTOF® was performed with MCF-7/182R-6 (C) and MDA-MB-436 (D) cells after DMSO and Olaparib (10 µM) treatment for 24 h and staining for EpCAM, CK, Vimentin, Cyclin A, 53BP1, pRPA32, γH2AX and RAD51. The fractions of EpCAM⁺, CK⁺ and Vimentin⁺ cells are given. For Cyclin A⁺, 53BP1⁺ and pRPA32⁺ cells, the fold changes are given. Mean values from DMSO-treated samples were set to 1 (absolute mean percentages for Cyclin A⁺ MCF-7/182R-6 cells: 19.2 %, MDA-MB-436 cells: 6.2 %, for 53BP1⁺ MCF-7/182R-6 cells: 59.8 %, MDA-MB-436 cells: 16.9 % and for pRPA32⁺ MCF-7/182R-6 cells: 11.2 %, MDA-MB-436 cells: 5.1 %). Boxes, intraquartile range; horizontal lines, median; plus, mean; whiskers, min to max. Significances were calculated by Wilcoxon test using GraphPad Prism 9. *:P<0.05. N=6-7. Exemplary dot plots are shown.

(A)

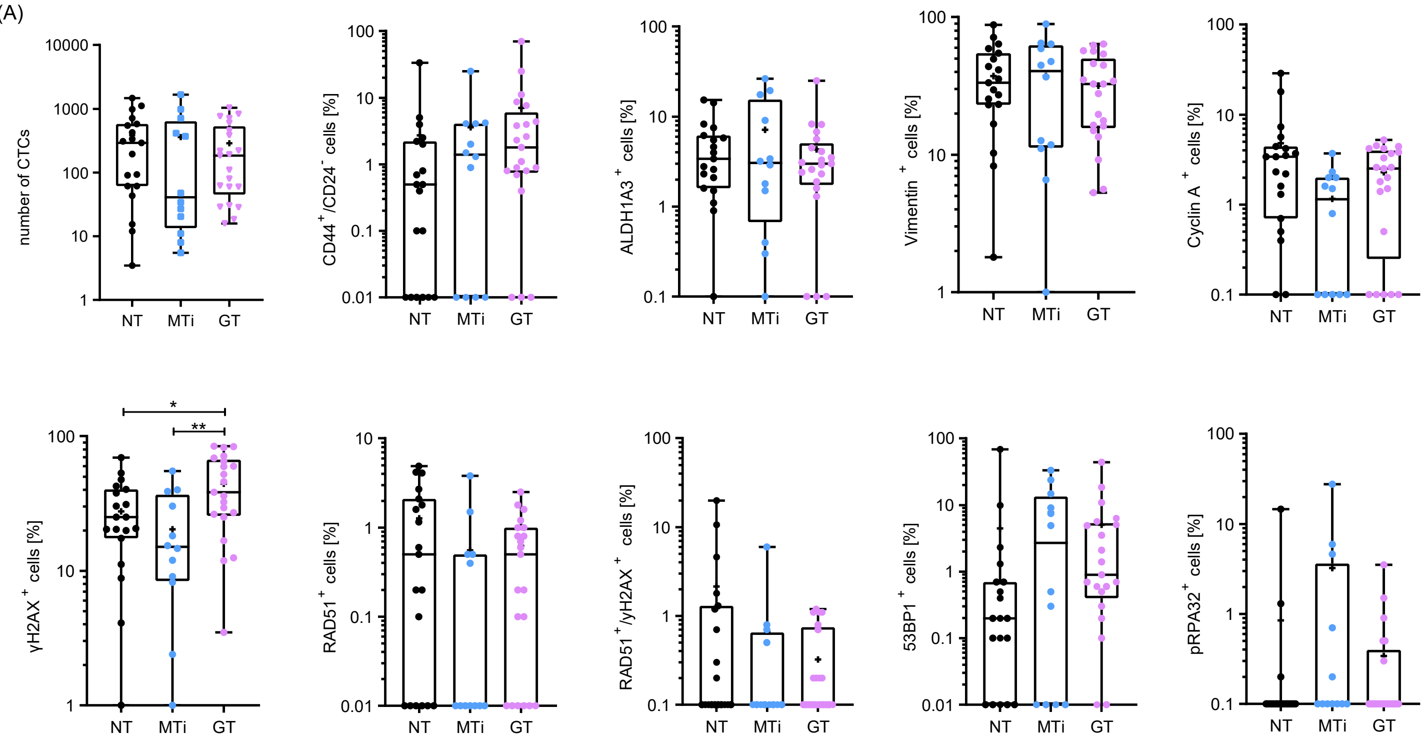

(B)

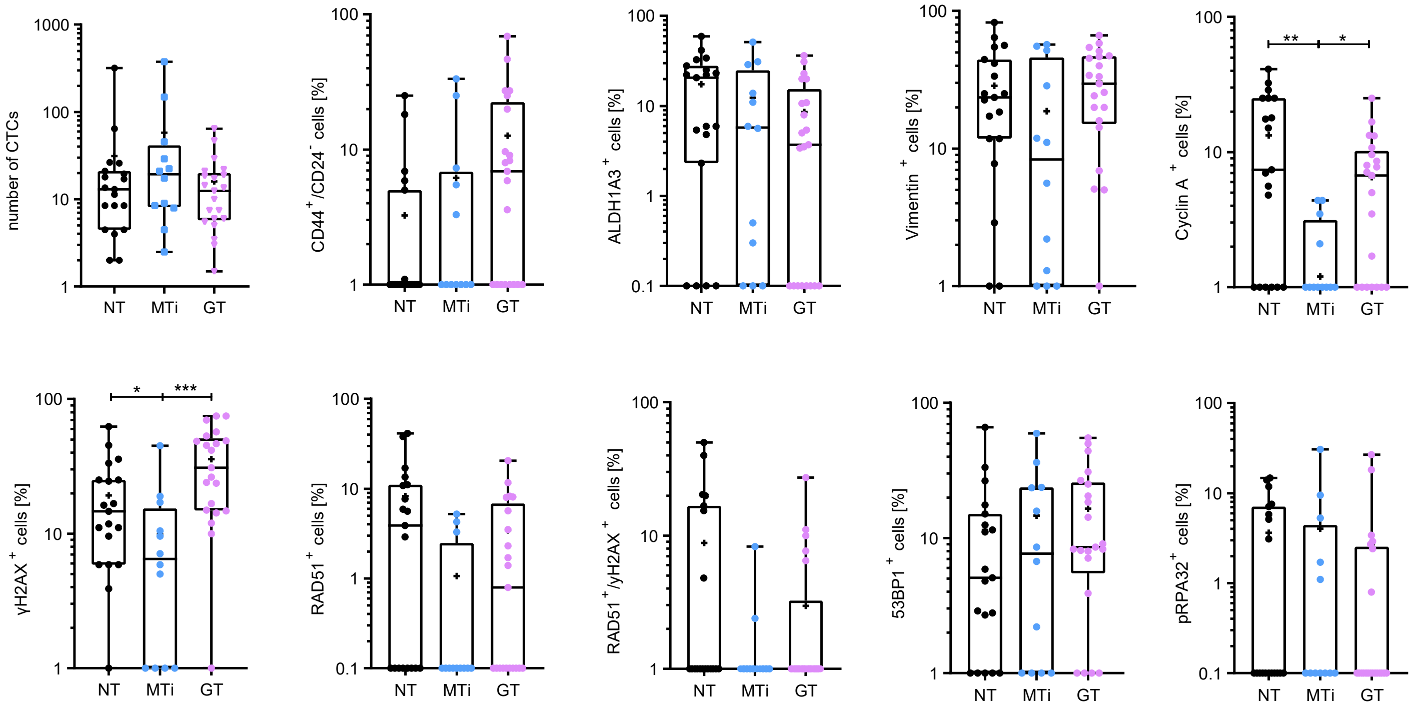

Supplementary Figure 2: Analysis of CTC numbers and biomarker positivities under different treatment lines. CyTOF®-based enumeration of $CD45^-$/$EpCAM^+$ (A) and $CD45^-$/CK^+ (B) CTCs/7.5 ml blood from mBC patients undergoing no treatment (NT), microtubule inhibitor treatment (MTi) or genotoxic treatment (GT), as well as further CTC characterization regarding the fractions of $CD44^+$/$CD24^-$, $ALDH1A3^+$, $Vimentin^+$, Cyclin A^+, $\gamma H2AX^+$, $RAD51^+$, $RAD51^+$/$\gamma H2AX^+$, $53BP1^+$ and $pRPA32^+$ CTCs in percent. Boxes, intraquartile range; horizontal lines, median; cross, mean; whiskers, min to max. Significances were calculated by Mann-Whitney U test, in case of statistical significance being reached with Kruskal-Wallis H-test, using GraphPad Prism 9. *: $P<0.05$, **: $P<0.01$, ***: $P<0.001$

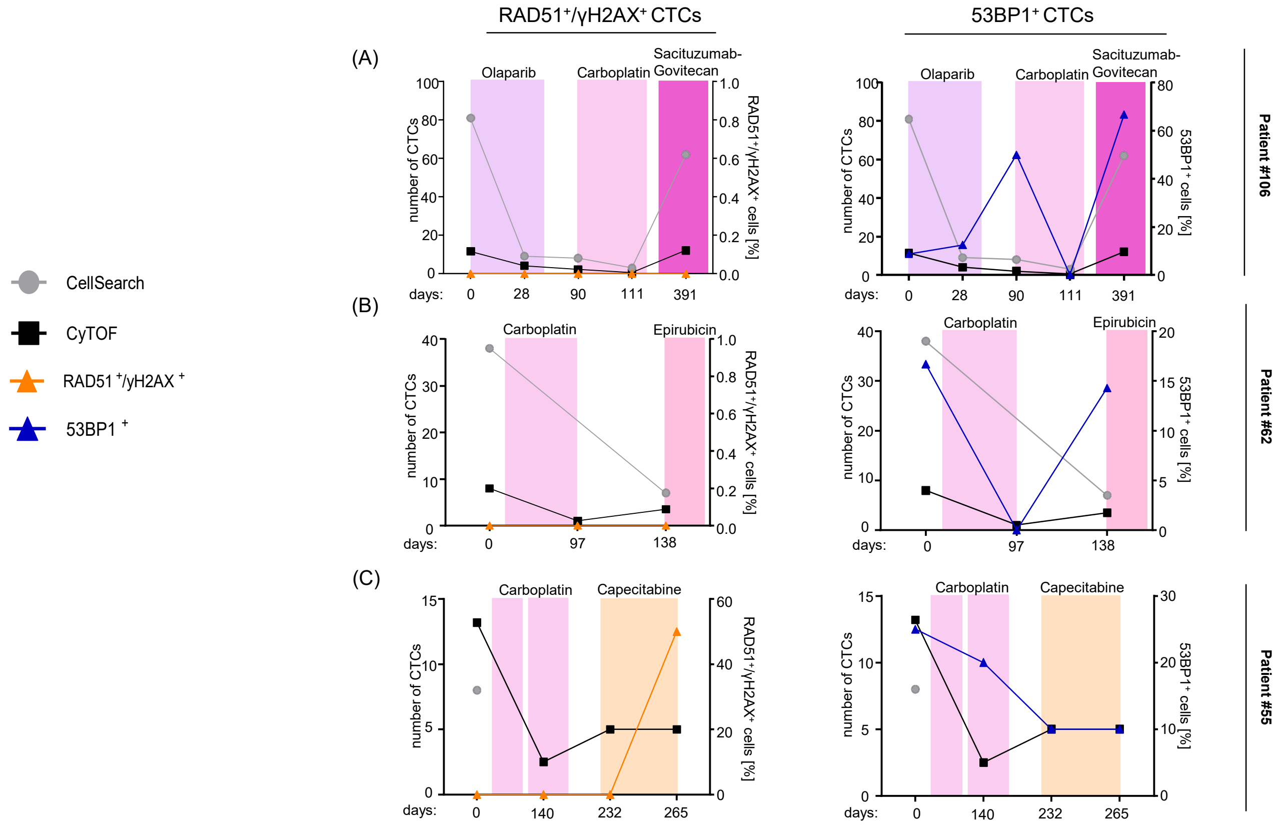

Supplementary Figure 3: The numbers of CTCs/7.5 ml blood identified by CellSearch® (grey) or CyTOF® (black) are graphically displayed as well as the percentages of RAD51⁺/γH2AX⁺ (orange) and 53BP1⁺ (blue) CTCs identified by CyTOF®. Shown are data for samples from patients #106 (A), #62 (B) and #55 (C)

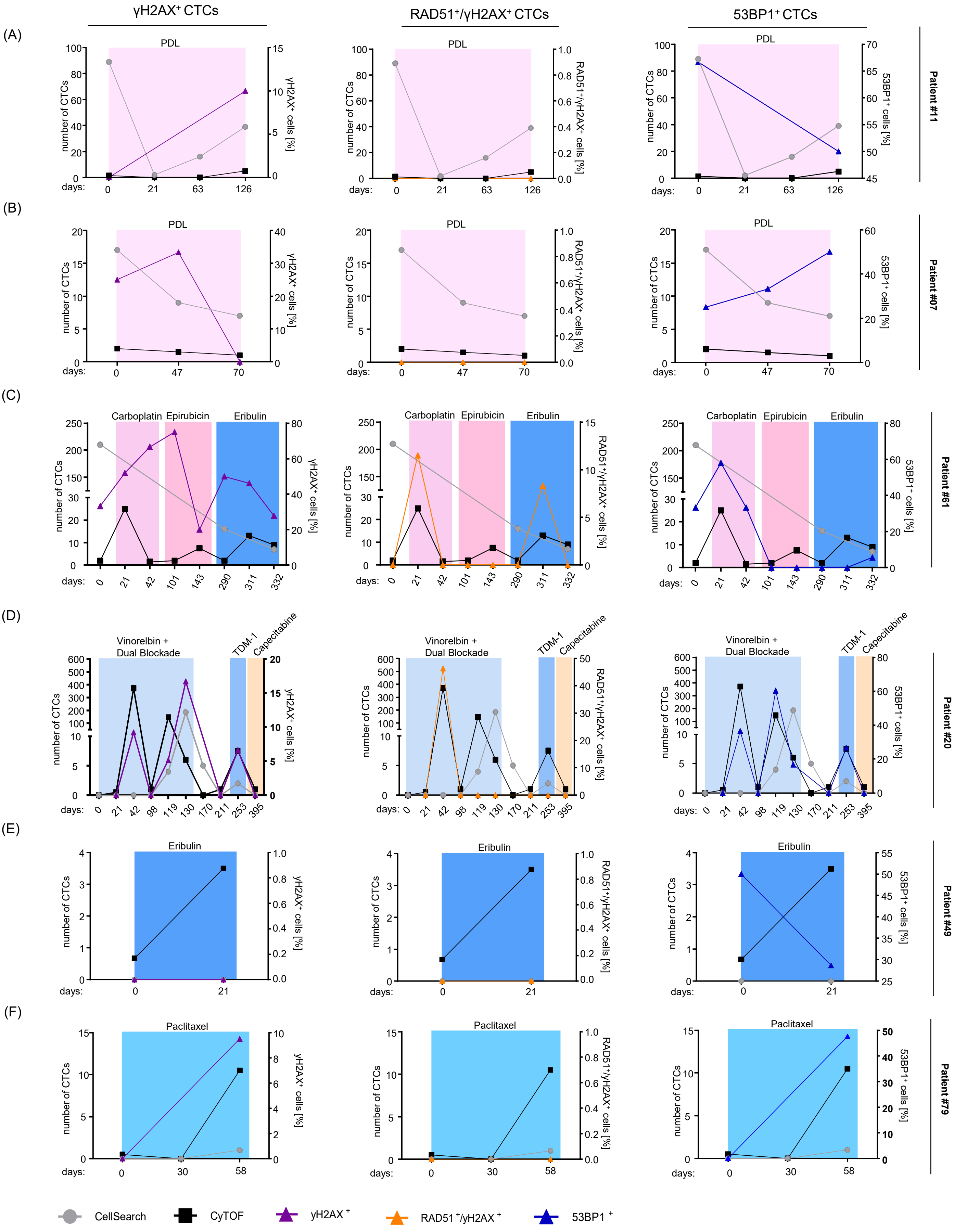

Supplementary Figure 4: The numbers of CTCs/7.5 ml blood identified by CellSearch® (grey) or CyTOF® (black) are graphically displayed as well as the percentages of γH2AX⁺ (purple), RAD51⁺/γH2AX⁺ (orange) and 53BP1⁺ (blue) CTCs identified by CyTOF®. Shown are data for samples from patients #11 (A), #07 (B), #61 (C), #20 (D), #49 (E) and #79 (F). PLD: Pegylated Liposomal Doxorubcin

(A)

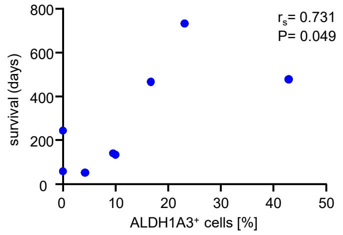

(B)

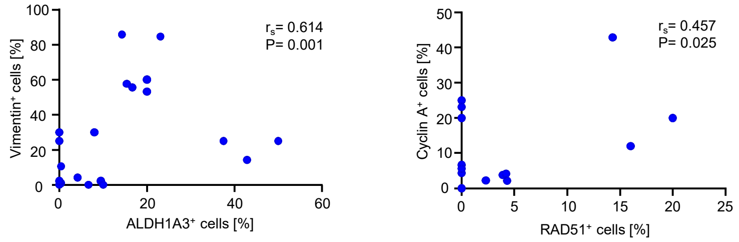

Supplementary Figure 5: Correlation of biomarker positivities with survival of the mBC patients. (A) Shown are Spearman correlations between the survival periods, given in days, and the corresponding percentages of ALDH1A3$^+$ CTCs. Survival periods were calculated starting from the date of the last analyzed sample to the date of death for each patient. (B) Correlations of Vimentin$^+$ with ALDH1A3$^+$ CTC fractions as well as of RAD51$^+$ with Cyclin A$^+$ CTC fractions. For all correlations Spearman's rank correlation coefficients and P values were calculated. Only samples which contained ≥3 CTCs/15 ml blood were included. Note that RAD51-positivity showed a trendwise correlation with survival as indicated in Table 2.

Supplementary references

1. Schochter F, Werner K, Köstler C, Faul A, Tzschaschel M, Alberter B, Müller V, Neubauer H, Fehm T, Friedl TWP, Polzer B, Janni W, Rack B, Wiesmüller L. 53BP1 Accumulation in Circulating Tumor Cells Identifies Chemotherapy-Responsive Metastatic Breast Cancer Patients. *Cancers (Basel)*. 2020;12(4):930. doi:10.3390/cancers12040930

2. Önder CE, Ziegler TJ, Becker R, Brucker SY, Hartkopf AD, Engler T, Koch A. Advancing Cancer Therapy Predictions with Patient-Derived Organoid Models of Metastatic Breast Cancer. *Cancers (Basel)*. 2023;15(14):3602. doi:10.3390/cancers15143602

3. Telli ML, Timms KM, Reid J, Hennessy B, Mills GB, Jensen KC, Szallasi Z, Barry WT, Winer EP, Tung NM, Isakoff SJ, Ryan PD, Greene-Colozzi A, Gutin A, Sangale Z, Iliev D, Neff C, Abkevich V, Jones JT, Lanchbury JS, Hartman AR, Garber JE, Ford JM, Silver DP, Richardson AL. Homologous Recombination Deficiency (HRD) Score Predicts Response to Platinum-Containing Neoadjuvant Chemotherapy in Patients with Triple-Negative Breast Cancer. *Clinical Cancer Research*. 2016;22(15):3764-3773. doi:10.1158/1078-0432.CCR-15-2477
